# Supplementary material for: Nickel-Imidazolium Low Transition Temperature Mixtures with Lewis-Acidic Character
Source: Molecules. 2023 Aug 30;28(17):6338. doi: 10.3390/molecules28176338 (PMC10490159; doi:10.3390/molecules28176338)
Supplement: Supplementary file 1 [file molecules-28-06338-s001.zip › molecules-2552502-supplementary.pdf]

molecules

Article

Nickel-imidazolium low transition temperature mixtures with Lewis-acidic character

Mario Martos [1], and Isidro M. Pastor [1,*]

[1] Organic Chemistry Department and Institute of Organic Synthesis (ISO), University of Alicante, ctra. San Vicente del Raspeig s/n, 03690, San Vicente del Raspeig, Alicante, Spain
* Correspondence: ipastor@ua.es; Tel.: (+34)965903728

Supplementary Information

Contents

Sustainability metrics 2
Differential Scanning Calorimetry traces 9
XPS analyses 12
NMR spectra 14
Miscellaneous images 26

molecules

MDPI

Sustainability metrics

Equations and general considerations

The sustainability metrics used (E-factor, yield, stoichiometric factor, atom economy, Andraos' reaction mass efficiency, materials recovery parameter) were calculated with equations 1-6:

$$\text{E–factor} = \frac{\sum \text{Mass of components} - \text{Mass of product}}{\text{Mass of product}} \tag{S1}$$

$$\text{Y} = \frac{\text{Mass of product obtained}}{\text{Theoretical mass of product}} \cdot 100 \tag{S2}$$

$$\text{AE} = \frac{\text{MW}_{\text{Prod}}}{\sum (\text{S}_{\text{Reactants}} \cdot \text{MW}_{\text{Reactants}})} \cdot 100 \tag{S3}$$

$$\text{SF} = \frac{\text{Mass of product}}{\text{Mass of stoichiometric reagents}} \cdot 100 \tag{S4}$$

$$\text{RME}_{\text{Andraos}} = \frac{\text{Mass of product}}{\sum \text{Mass of components} - \sum \text{Mass recovered}} \cdot 100 \tag{S5}$$

$$\text{MRP} = \frac{1}{(1+(\frac{\text{Mass of product}}{\sum \text{Mass of reagents}} \cdot 100) \cdot \text{E}_{\text{Aux}})} \cdot 100 \tag{S6}$$

The Y, AE, SF, $\text{RME}_{\text{Andraos}}$ and MRP values were combined into a vector magnitude ratio (VMR) using equation 7:

$$\text{VMR} = \frac{1}{\sqrt{5}} \sqrt{(\text{Y})^2 + (\text{AE})^2 + (\text{RME})^2 + \left(\frac{1}{\text{SF}}\right)^2 + (\text{MRP})^2} \tag{S7}$$

The EcoScale score was obtained using the tool developed by the authors, available at http://ecoscale.cheminfo.org/calculator (last accessed July 24th 2023)

Some additional considerations and standard values applied to the calculations are compiled in Table S1.

Table S1. Standard values for the calculation of sustainability metrics on protocols with missing data.

Parameter	Value
Solvent recovery (reaction, work-up)	80% of the initial mass if not requiring fractional distillation
Water and saturated sodium chloride for work-up	Equal volume to the first fraction of organic solvent used
Work-up reported but not specified	3x10 mL/mmol of ethyl acetate and 10 mL/mmol of saturated sodium chloride
Drying agents	1/5th of the total mass of organic solvent

Full metrics of the comparison of Table 2

Table S2. Sustainability metrics for the synthesis of 1. Entries correlate with those of Table 2.

Entry	Yield	1/SF	AE	RME	MRP	E-factor	EcoScale
**1**	0.99	1	0.945	0.201	0.212	3.98	79
**2**	0.99	1	0.945	0.345	0.369	1.90	89
**3**	0.88	0.735	0.945	0.054	0.087	17.59	70
**4**	0.96	0.847	0.945	0.422	0.549	1.37	77
**5**	0.95	1	0.945	0.01	0.012	94.82	79
**6**	0.96	1	0.945	0.003	0.003	360.36	69

Purification by chromatography omitted for RME, MRP and E-factor in entries 3 to 6.

				Parameter	Actual	Ideal Limit
Actual Mass of Reagents		0,0817	g	AE	0,945	1
Stoichiometric Mass of Reagents		0,08	g	Rxn Yield	0,998	1
Stoichiometric Factor		1,001097309		1/SF	0,999	1
Materials Recovery Parameter		0,212926766		MRP	0,212926766	1
				RME	0,201	1
Raw E-factor profile						
E-kernel		0,05987595				
E-excess		0,001163011		VMR	0,771141135	
E-rxn solvent		0		(vector magnitude ratio)		
E-catalyst		0,064935065				
E-work-up		0				
E-purification		12,36363636				
	E-aux	12,43				
E-total		12,48961039				
**Corrected E-factor**		**3,983**				
Yield		99,8	%			
AE		94,5	%			
PMI		4,983				
RME (global)		20,1	%			

Figure S1. Sustainability metrics for the synthesis of 1 (Table 2, entry 1).

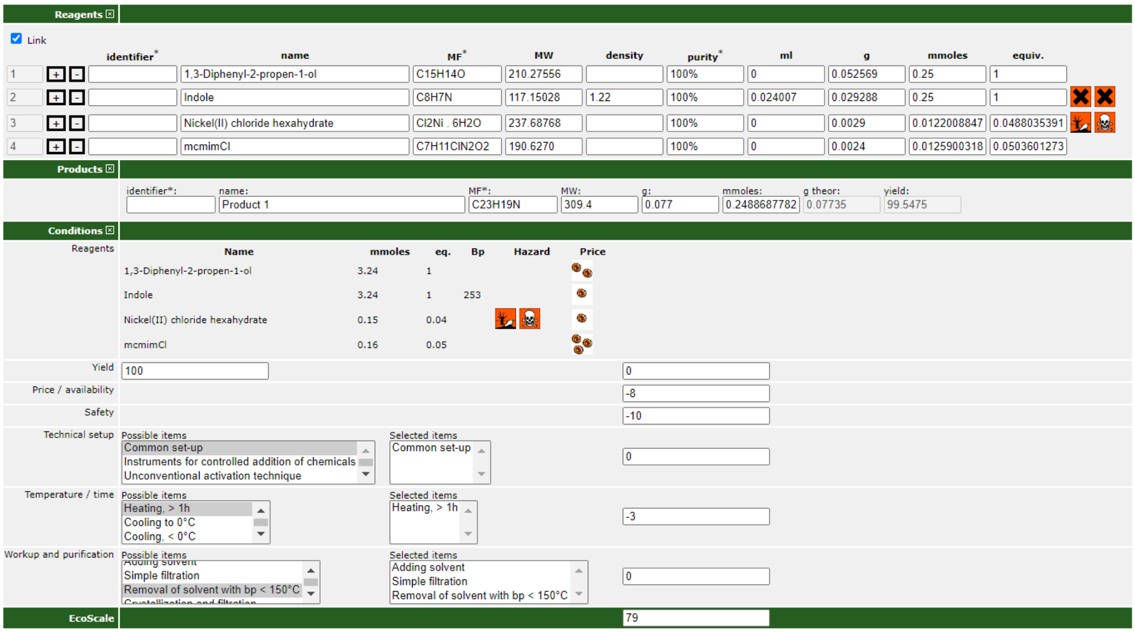

Figure S2. EcoScale for the synthesis of 1 (Table 2, entry 1).

Actual Mass of Reagents		1,65	g	**Parameter**	**Actual**	**Ideal Limit**
Stoichiometric Mass of Reagents		1,65	g	AE	0,945	1
Stoichiometric Factor		1,000619636		Rxn Yield	0,988	1
Materials Recovery Parameter		0,369127517		1/SF	0,999	1
				MRP	0,369127517	1
Raw E-factor profile				RME	0,345	1
E-kernel		0,070765086				
E-excess		0,000663485		VMR	0,790368533	
E-rxn solvent		0		(vector magnitude ratio)		
E-catalyst		0,071428571				
E-work-up		0				
E-purification		6,506493506				
	E-aux	6,58				
E-total		6,649350649				
**Corrected E-factor**		**1,903**				
Yield		98,8	%			
AE		94,5	%			
PMI		2,903				
RME (global)		34,5	%			

Figure S3. Sustainability metrics for the synthesis of 1 (Table 2, entry 2).

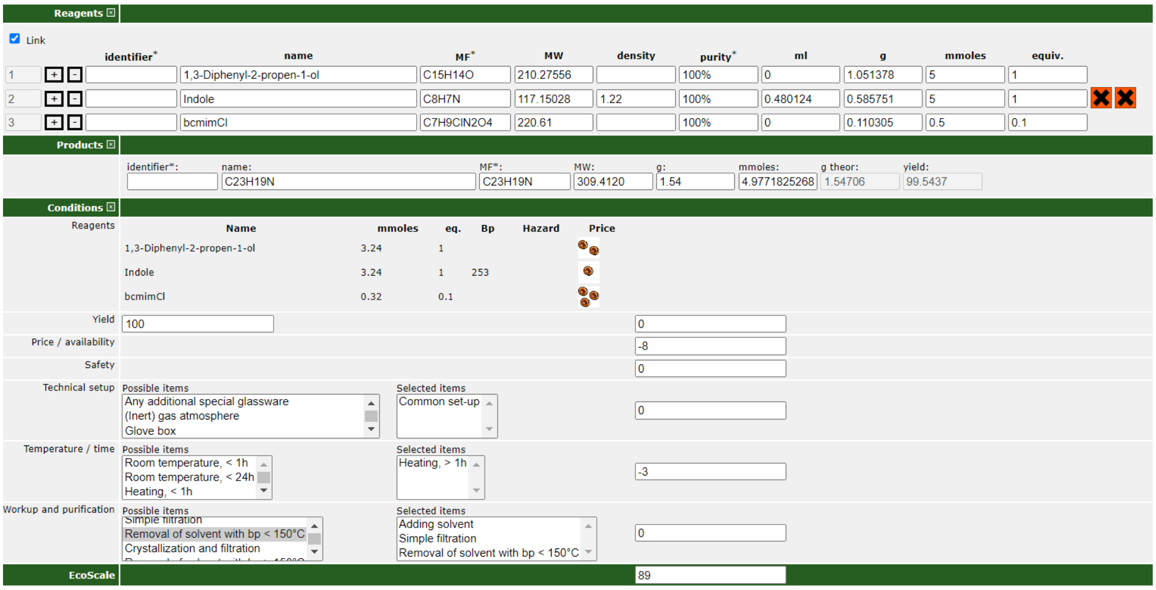

Reagents

Link

	identifier*	name	MF*	MW	density	purity*	ml	g	mmoles	equiv.
1		1,3-Diphenyl-2-propen-1-ol	C15H14O	210.27556		100%	0	1.051378	5	1
2		Indole	C8H7N	117.15028	1.22	100%	0.480124	0.585751	5	1
3		bcmimCl	C7H9ClN2O4	220.61		100%	0	0.110305	0.5	0.1

Products

identifier*:	name:	MF*:	MW:	g:	mmoles:	g theor:	yield:
	C23H19N	C23H19N	309.4120	1.54	4.9771825268	1.54706	99.5437

Conditions

Reagents

Name	mmoles	eq.	Bp	Hazard	Price
1,3-Diphenyl-2-propen-1-ol	3.24	1			
Indole	3.24	1	253		
bcmimCl	0.32	0.1			

	Possible items	Selected items	
Yield	100		0
Price / availability			-8
Safety			0
Technical setup	Any additional special glassware; (Inert) gas atmosphere; Glove box	Common set-up	0
Temperature / time	Room temperature, < 1h; Room temperature, < 24h; Heating, < 1h	Heating, > 1h	-3
Workup and purification	Simple filtration; Removal of solvent with bp < 150°C; Crystallization and filtration	Adding solvent; Simple filtration; Removal of solvent with bp < 150°C	0
**EcoScale**			89

Figure S4. EcoScale for the synthesis of 1 (Table 2, entry 2).

Actual Mass of Reagents		0,222	g	Parameter	Actual	Ideal Limit
Stoichiometric Mass of Reagents		0,16	g	AE	0,945	1
Stoichiometric Factor		1,357799942		Rxn Yield	0,887	1
Materials Recovery Parameter		0,087127159		1/SF	0,736	1
				MRP	0,087127159	1
Raw E-factor profile				RME	0,054	1
E-kernel		0,193429095				
E-excess		0,427008861		VMR	0,668150897	
E-rxn solvent		7,299270073		(vector magnitude ratio)		
E-catalyst		0,102189781				
E-work-up		46,80291971				
E-purification		0				
	E-aux	54,20				
E-total		54,82481752				
**Corrected E-factor**		**17,599**				
Yield		88,7	%			
AE		94,5	%			
PMI		18,599				
RME (global)		5,4	%			

Figure S5. Sustainability metrics for the synthesis of 1 (Table 2, entry 3).

Reagents

Link

	identifier*	name	MF*	MW	density	purity*	ml	g	mmoles	equiv.
1		1,3-Diphenyl-2-propen-1-ol	C15H14O	210.27556		100%	0	0.105138	0.5000000000	1
2		Indole	C8H7N	117.15028	1.22	100%	0.096025	0.11715	1.0000000000	2
3		Iron(III) chloride hexahydrate	H12O6Cl3Fe	270.29768		100%	0	0.013515	0.0500000000	0.1
4		Water	H2O	18.01528	1	100%	10	10	555.08435061	1110.1687012

Products

identifier*:	name:	MF*:	MW:	g:	mmoles:	g theor:	yield:
	C23H19N	C23H19N	309.412	0.137	0.4427753286	0.154706	88.5551

Conditions

Reagents

Name	mmoles	eq.	Bp	Hazard	Price
1,3-Diphenyl-2-propen-1-ol	3.64	1			
Indole	7.29	2	253		
Iron(III) chloride hexahydrate	0.36	0.1			
Water	4051.71	1110.16			

	Possible items	Selected items	
Yield	89		-6
Price / availability			-8
Safety			0
Technical setup	Any additional special glassware<br>(Inert) gas atmosphere<br>Glove box	Common set-up	0
Temperature / time	Heating, > 1h<br>Cooling to 0°C<br>Cooling, < 0°C	Heating, > 1h	-3
Workup and purification	Simple filtration<br>Removal of solvent with bp < 150°C<br>Crystallization and filtration	Liquid - liquid extraction or washing<br>Classical chromatography<br>Removal of solvent with bp < 150°C	-13
**EcoScale**			70

Figure S6. EcoScale for the synthesis of 1 (Table 2, entry 3).

Actual Mass of Reagents		0,193	g	Parameter	Actual	Ideal Limit
Stoichiometric Mass of Reagents		0,16	g	AE	0,945	1
Stoichiometric Factor		1,18042968		Rxn Yield	0,958	1
Materials Recovery Parameter		0,54985755		1/SF	0,847	1
				MRP	0,54985755	1
Raw E-factor profile				RME	0,422	1
E-kernel		0,104728284				
E-excess		0,19932577		VMR	0,77567565	
E-rxn solvent		5,391891892		(vector magnitude ratio)		
E-catalyst		0				
E-work-up		0				
E-purification		0				
	E-aux	5,39				
E-total		5,695945946				
**Corrected E-factor**		**1,372**				
Yield		95,8	%			
AE		94,5	%			
PMI		2,372				
RME (global)		42,2	%			

Figure S7. Sustainability metrics for the synthesis of 1 (Table 2, entry 4).

Reagents

Link

	identifier*	name	MF*	MW	density	purity*	ml	g	mmoles	equiv.
1		1,3-Diphenyl-2-propen-1-ol	C15H14O	210.27556		100%	0	0.105138	0.5	1
2		Indole	C8H7N	117.15028	1.22	100%	0.072019	0.087863	0.75	1.5
3		1,1,1,3,3,3-Hexafluoro-2-propanol	C3H2F6O	168.038698	1.596	100%	0.5	0.798	4.7489061120	9.4978122241

Products

identifier*:	name:	MF*:	MW:	g:	mmoles:	g theor:	yield:
	C23H19N	C23H19N	309.421	0.148	0.4783127195	0.154711	95.6622

Conditions

Reagents

Name	mmoles	eq.	Bp	Hazard	Price
1,3-Diphenyl-2-propen-1-ol	3.37	1			
Indole	5.06	1.5	253		
1,1,1,3,3,3-Hexafluoro-2-propanol	32.08	9.49	59		

	Possible items	Selected items	
Yield	96		-2
Price / availability			-8
Safety			0
Technical setup	Common set-up; Instruments for controlled addition of chemicals; Unconventional activation technique	Common set-up	0
Temperature / time	Heating, > 1h; Cooling to 0°C; Cooling, < 0°C	Heating, > 1h	-3
Workup and purification	Sublimation; Liquid - liquid extraction or washing; Classical chromatography	Classical chromatography	-10
**EcoScale**			77

Figure S8. EcoScale for the synthesis of 1 (Table 2, entry 4).

Actual Mass of Reagents		0,327 g	Parameter	Actual	Ideal Limit
Stoichiometric Mass of Reagents		0,33 g	AE	0,945	1
Stoichiometric Factor		1,000001309	Rxn Yield	0,951	1
Materials Recovery Parameter		0,011607476	1/SF	1,000	1
			MRP	0,011607476	1
Raw E-factor profile			RME	0,010	1
E-kernel		0,112243442			
E-excess		1,45585E-06	VMR	0,748124397	
E-rxn solvent		4,523809524	(vector magnitude ratio)		
E-catalyst		0,032312925			
E-work-up		90,15306122			
E-purification		0			
	E-aux	94,71			
E-total		94,82142857			
**Corrected E-factor**		**94,821**			
Yield		95,1 %			
AE		94,5 %			
PMI		95,821			
RME (global)		1,0 %			

Figure S9. Sustainability metrics for the synthesis of 1 (Table 2, entry 5).

Reagents

Link

	identifier*	name	MF*	MW	density	purity*	ml	g	mmoles	equiv.
1		1,3-Diphenyl-2-propen-1-ol	C15H14O	210.27556		100%	0	0.210276	1	1
2		Indole	C8H7N	117.15028	1.22	100%	0.096025	0.11715	1	1
3		p-Toluenesulfonic acid monohydrate	C7H8O3S . H2O	190.214		100%	0	0.009511	0.05	0.05
4		Dichloromethane	CH2Cl2	84.93288	1.325	100%	1	1.325	15.600554225	15.600554225

Products

identifier*:	name:	MF*:	MW:	g:	mmoles:	g theor:	yield:
	C23H19N	C23H19N	309.412	0.294	0.9501893914	0.309412	95.0189

Conditions

Reagents

Name	mmoles	eq.	Bp	Hazard	Price
1,3-Diphenyl-2-propen-1-ol	3.4	1			
Indole	3.4	1	253		
p-Toluenesulfonic acid monohydrate	0.17	0.05			
Dichloromethane	53.06	15.6	39		

	Possible items	Selected items	
Yield	95		-2
Price / availability			-3
Safety			0
Technical setup	Common set-up<br>Instruments for controlled addition of chemicals<br>Unconventional activation technique	Common set-up	0
Temperature / time	Heating, > 1h<br>Cooling to 0°C<br>Cooling, < 0°C	Heating, > 1h	-3
Workup and purification	None<br>Cooling to room temperature<br>Adding solvent	Liquid - liquid extraction or washing<br>Classical chromatography	-13
**EcoScale**			79

Figure S10. EcoScale for the synthesis of 1 (Table 2, entry 5).

Actual Mass of Reagents		0,0327 g		**Parameter**	**Actual**	**Ideal Limit**
Stoichiometric Mass of Reagents		0,03 g		AE	0,945	1
Stoichiometric Factor		1,000001309		Rxn Yield	0,961	1
Materials Recovery Parameter		0,00304682		1/SF	1,000	1
				MRP	0,00304682	1
Raw E-factor profile				RME	0,003	1
E-kernel		0,10100866				
E-excess		1,44115E-06		VMR	0,750572138	
E-rxn solvent		8,417508418		(vector magnitude ratio)		
E-catalyst		0				
E-work-up		624,3703704				
E-purification		91,11111111				
	E-aux	723,90				
E-total		724				
**Corrected E-factor**		**360,364**				
Yield		96,1 %				
AE		94,5 %				
PMI		361,364				
RME (global)		0,3 %				

Figure S11. Sustainability metrics for the synthesis of 1 (Table 2, entry 6).

Reagents

Link

	identifier*	name	MF*	MW	density	purity*	ml	g	mmoles	equiv.
1		1,3-Diphenyl-2-propen-1-ol	C15H14O	210.27556		100%	0	0.021028	0.1	1
2		Indole	C8H7N	117.15028	1.22	100%	0.009602	0.011715	0.1	1
3		DL-Lactic acid	C3H6O3	90.07884		100%	0	0.14	1.5541940815	15.541940815
4		Choline chloride	C5H14ClNO	139.62526		100%	0	0.11	0.7878230629	7.8782306296

Products

identifier*:	name:	MF*:	MW:	g:	mmoles:	g theor:	yield:
	Product 1	C23H19N	309.4	0.0297	0.0959922430	0.03094	95.992200000

Conditions

Reagents

Name	mmoles	eq.	Bp	Hazard	Price
1,3-Diphenyl-2-propen-1-ol	3.36	1			
Indole	3.36	1	253		
DL-Lactic acid	52.32	15.54			
Choline chloride	26.52	7.87			

	Possible items	Selected items	
Yield	96		-2
Price / availability			-13
Safety			0
Technical setup	Common set-up; Instruments for controlled addition of chemicals; Unconventional activation technique	Common set-up	0
Temperature / time	Heating, > 1h; Cooling to 0°C; Cooling, < 0°C	Heating, > 1h	-3
Workup and purification	Sublimation; Liquid - liquid extraction or washing; Classical chromatography	Adding solvent; Liquid - liquid extraction or washing; Classical chromatography	-13
**EcoScale**			69

Figure S12. EcoScale for the synthesis of 1 (Table 2, entry 6).

Differential Scanning Calorimetry traces

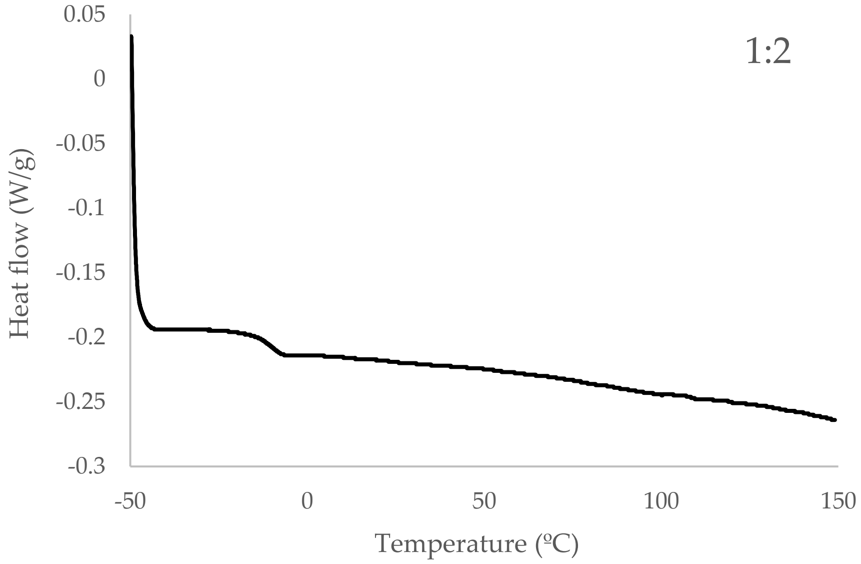

Figure S13. DSC trace of the 1:2 mcmimCl:$NiCl_2$ mixture (scanning rate 5 ºC/min)

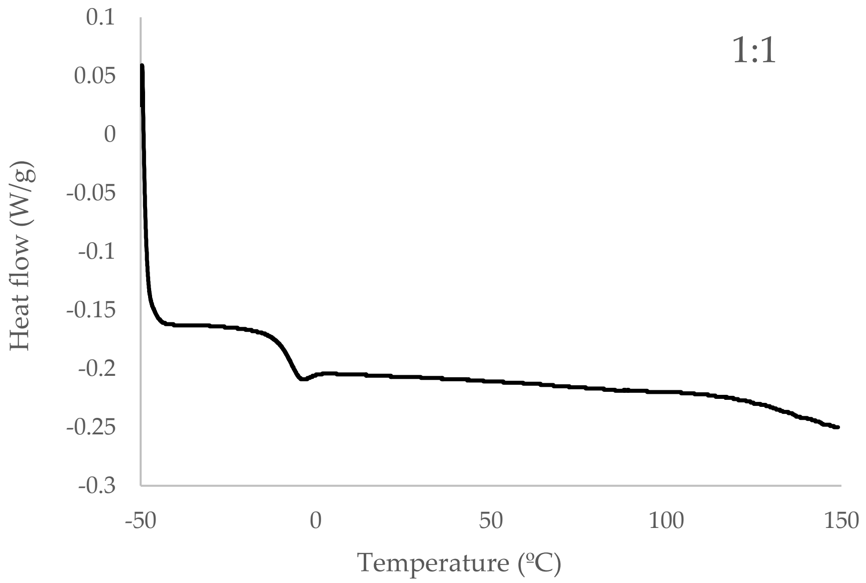

Figure S14. DSC trace of the 1:1 mcmimCl:$NiCl_2$ mixture (scanning rate 5 ºC/min)

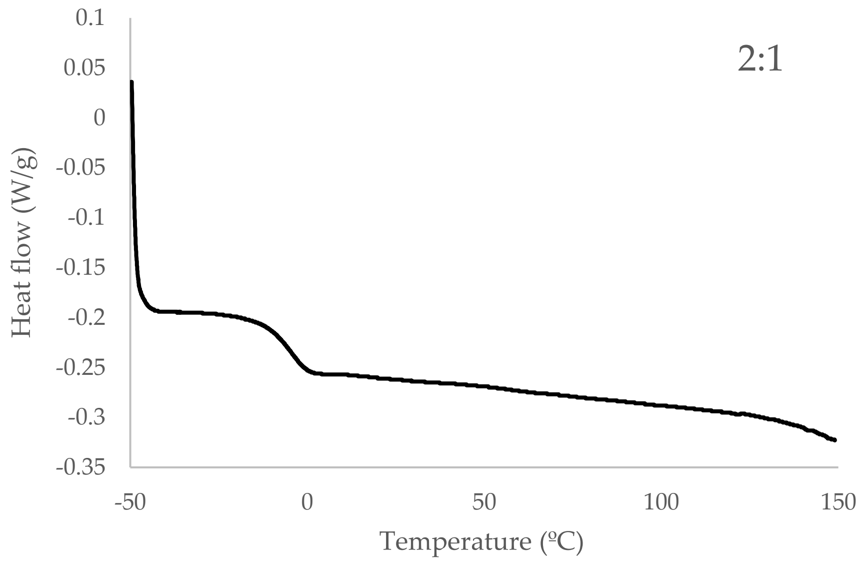

Figure S15. DSC trace of the 2:1 mcmimCl:$NiCl_2$ mixture (scanning rate 5 ºC/min)

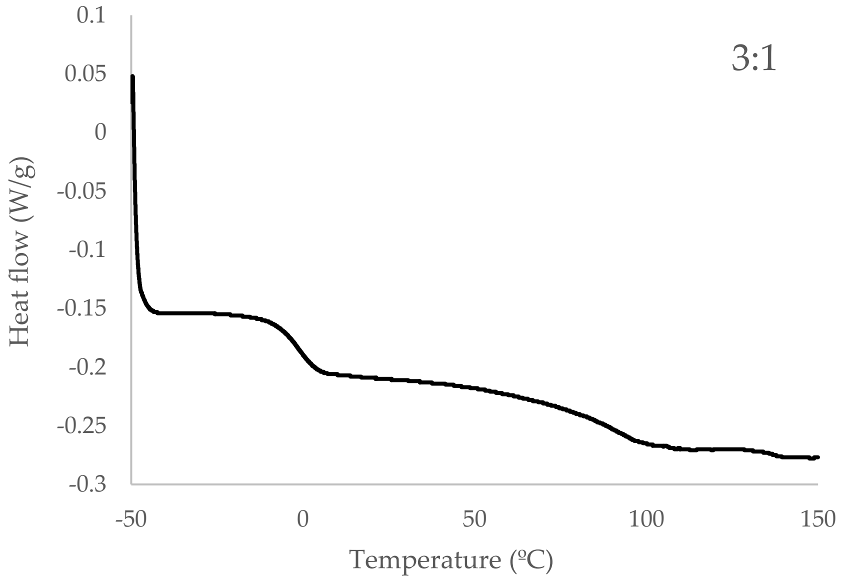

Figure S16. DSC trace of the 3:1 mcmimCl:$NiCl_2$ mixture (scanning rate 5 ºC/min)

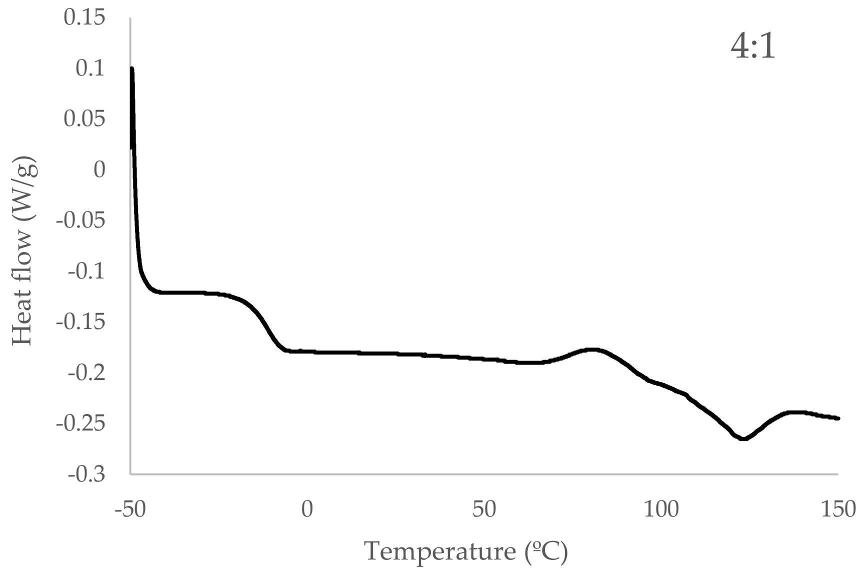

Figure S17. DSC trace of the 4:1 mcmimCl:$NiCl_2$ mixture (scanning rate 5 ºC/min)

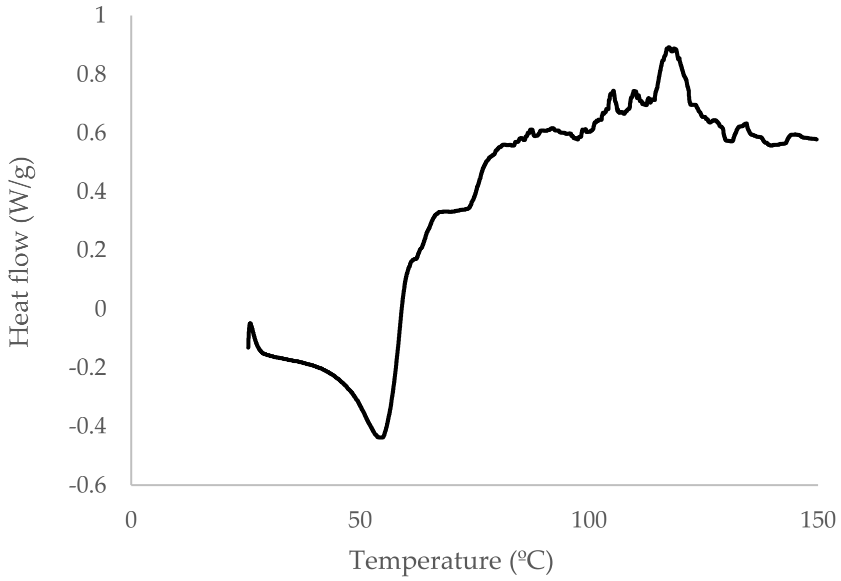

Figure S18. DSC trace of the 1:1 mcmimCl:$NiCl_2 \cdot 6H_2O$ mixture (scanning rate 5 ºC/min)

XPS analyses

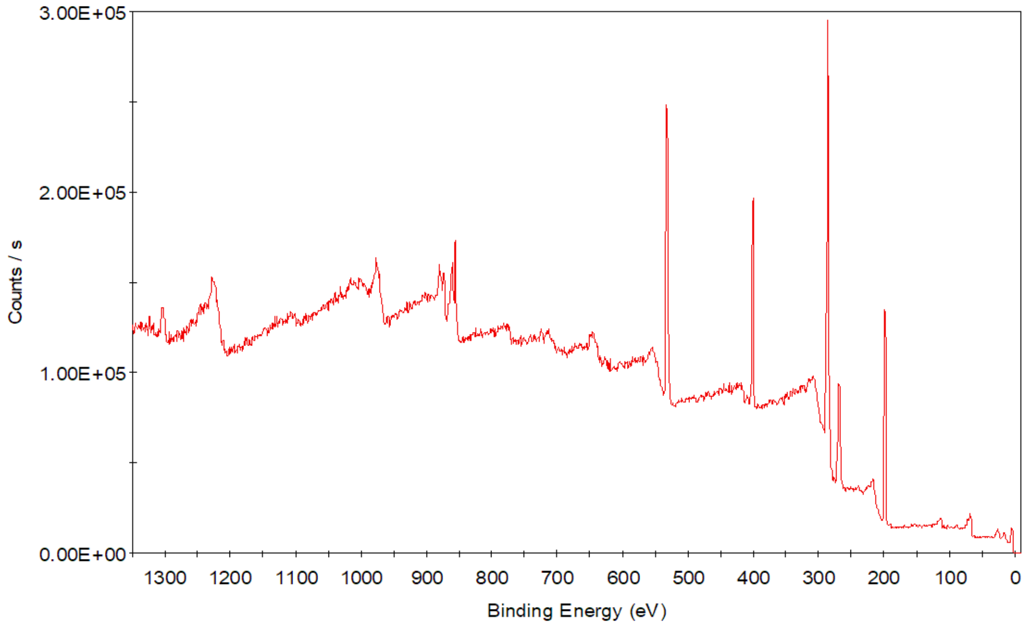

Figure S19. XPS survey of the 2:1 mcmimCl:$NiCl_2$ mixture

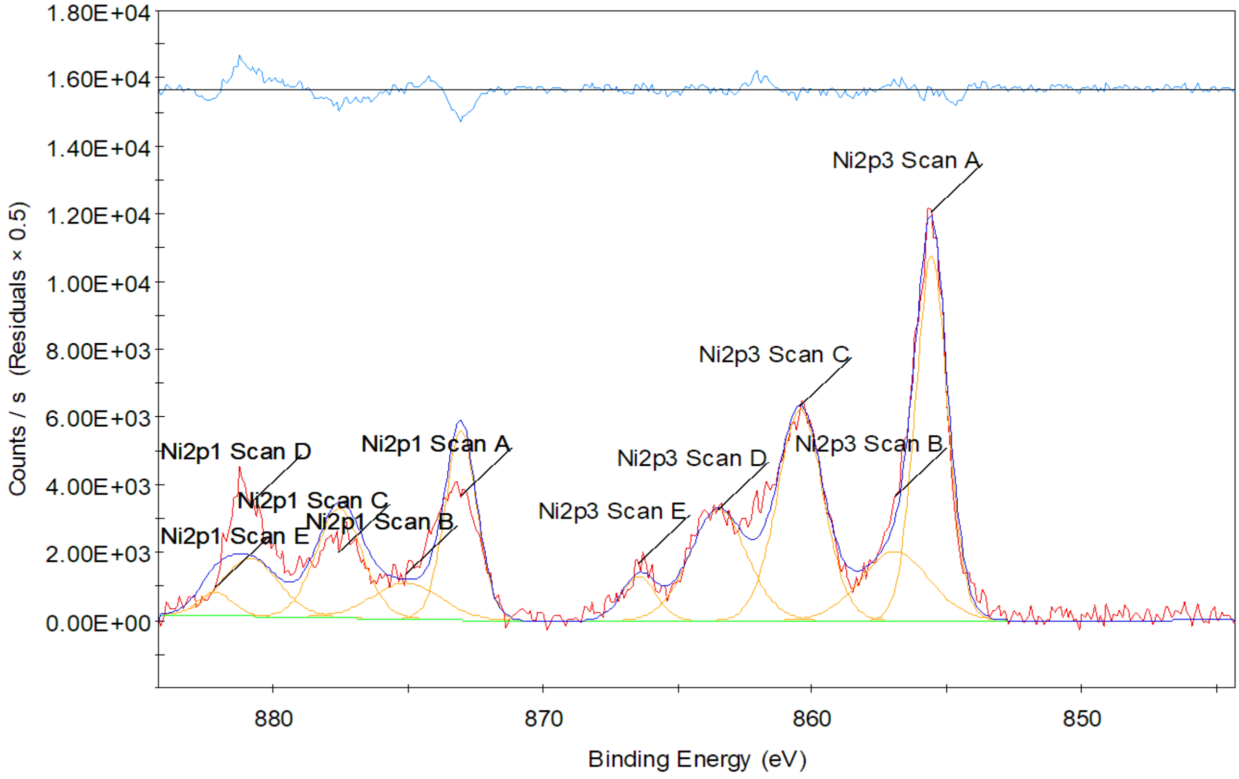

Figure S20. High resolution XPS Ni 2p scan of the 2:1 mcmimCl:$NiCl_2$ mixture

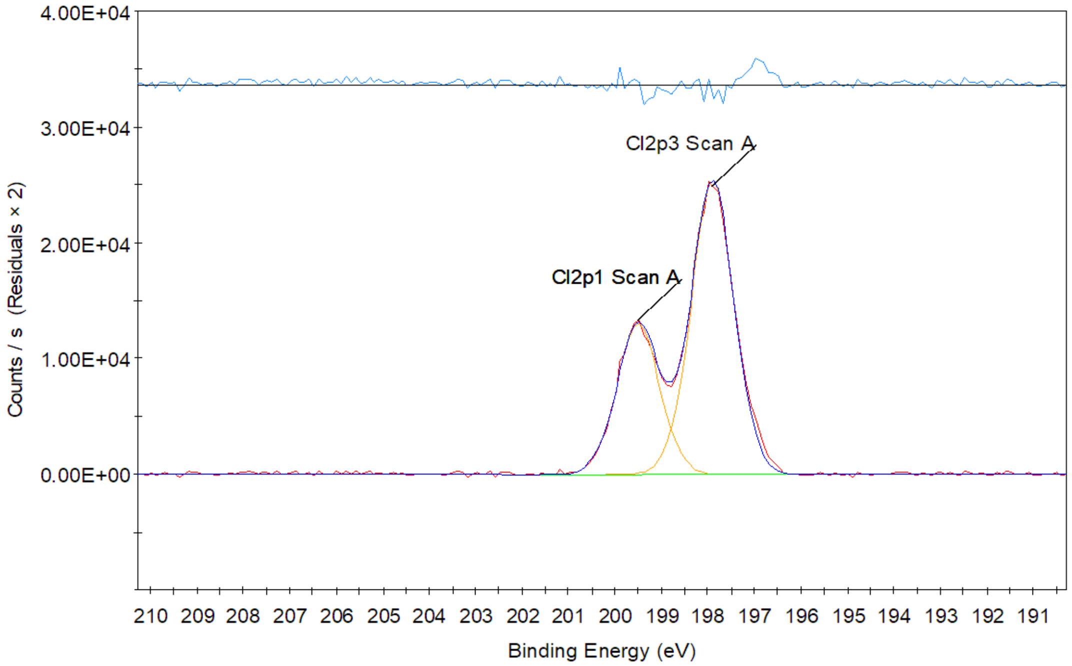

Figure S21. High resolution XPS Cl 2p scan of the 2:1 mcmimCl:$NiCl_2$ mixture

molecules

NMR spectra

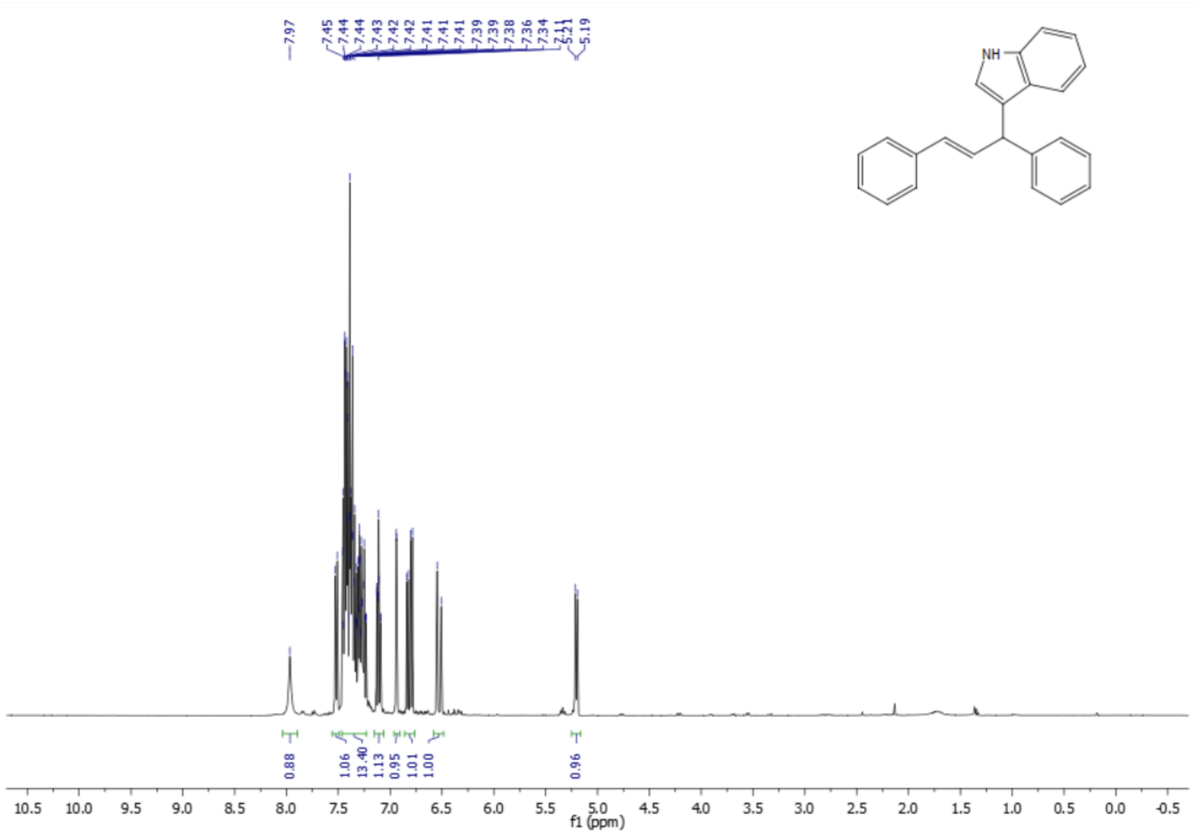

Figure S22. ^{1}H NMR spectrum of *(E)*-3-(1,3-diphenylallyl)-1*H*-indole (1)

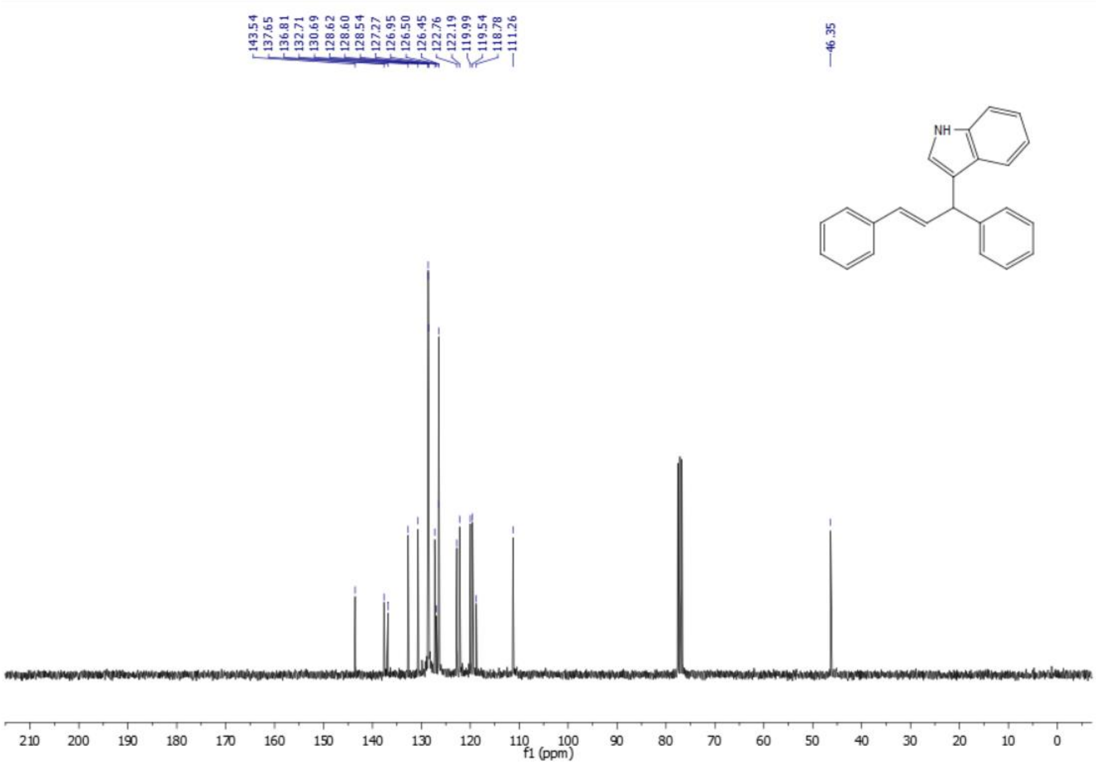

Figure S23. ^{13}C NMR spectrum of *(E)*-3-(1,3-diphenylallyl)-1*H*-indole (1)

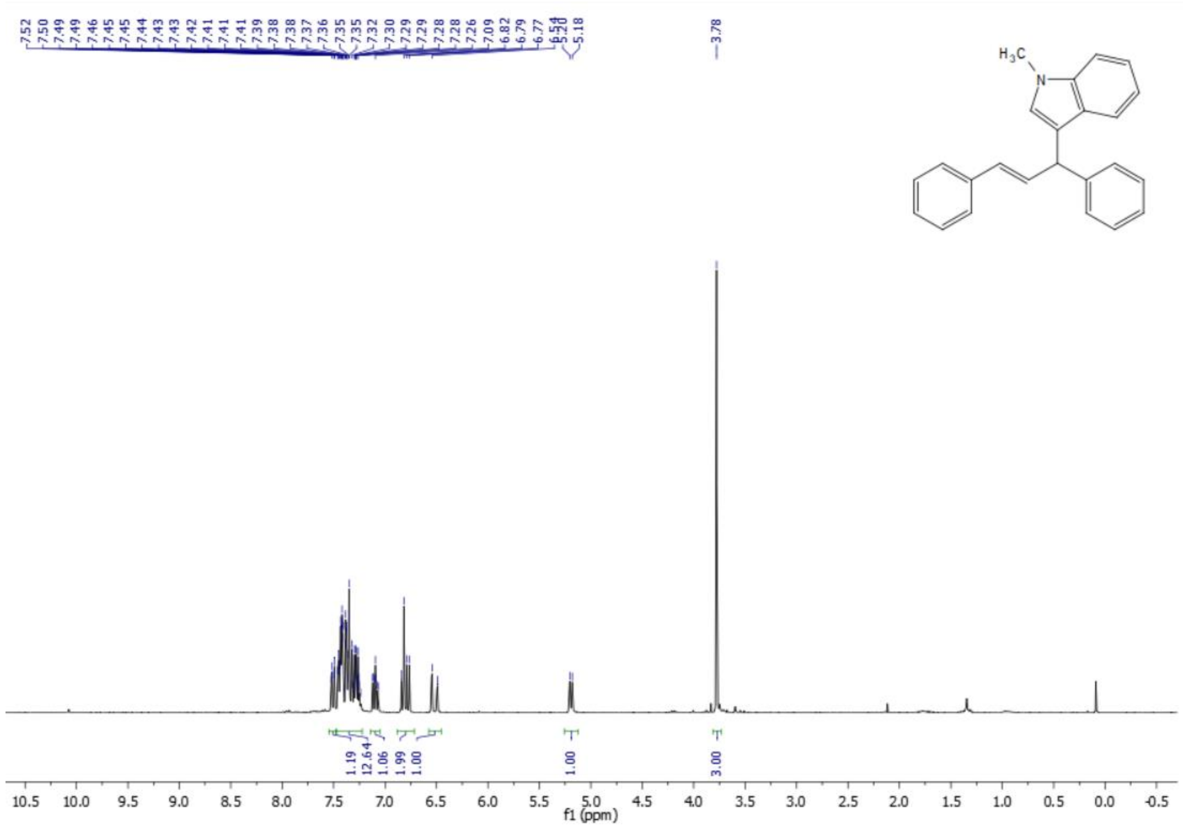

Figure S24. ^{1}H NMR spectrum of (*E*)-3-(1,3-diphenylallyl)-1-methyl-1*H*-indole (2)

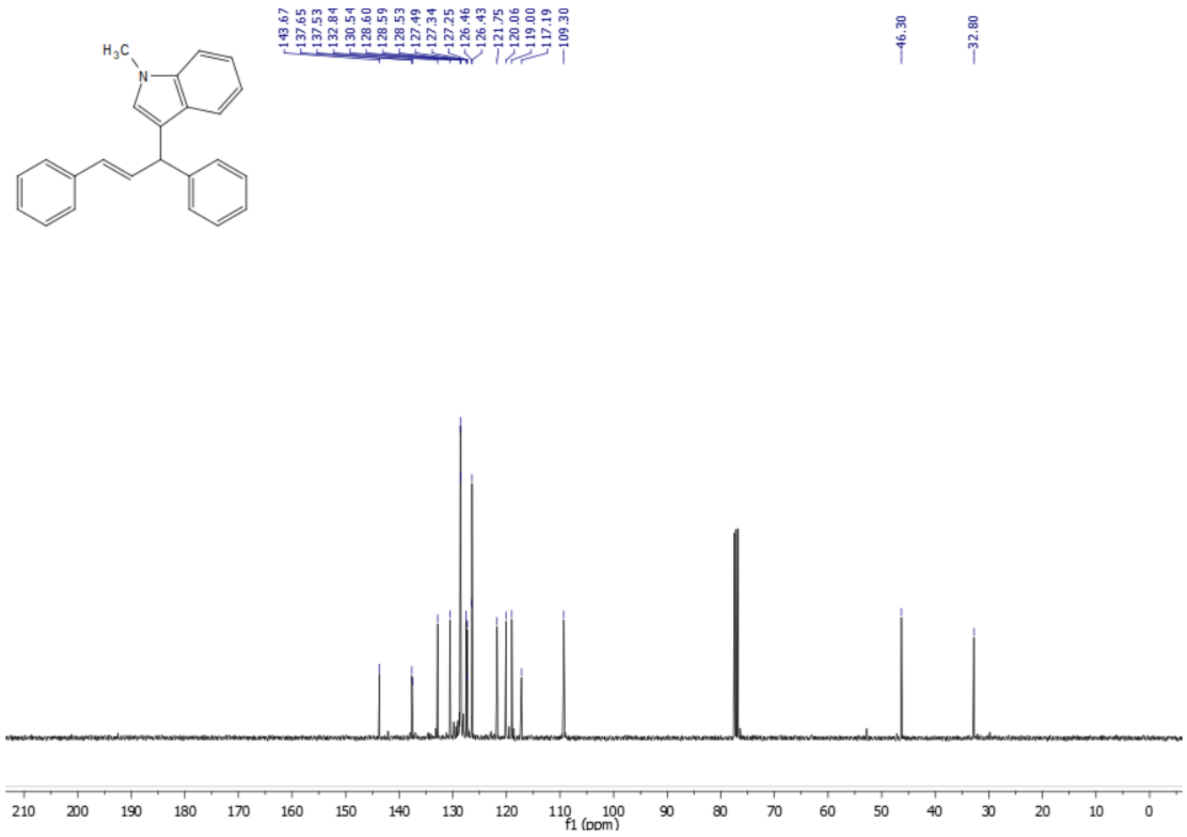

Figure S25. ^{13}C NMR spectrum of (*E*)-3-(1,3-diphenylallyl)-1-methyl-1*H*-indole (2)

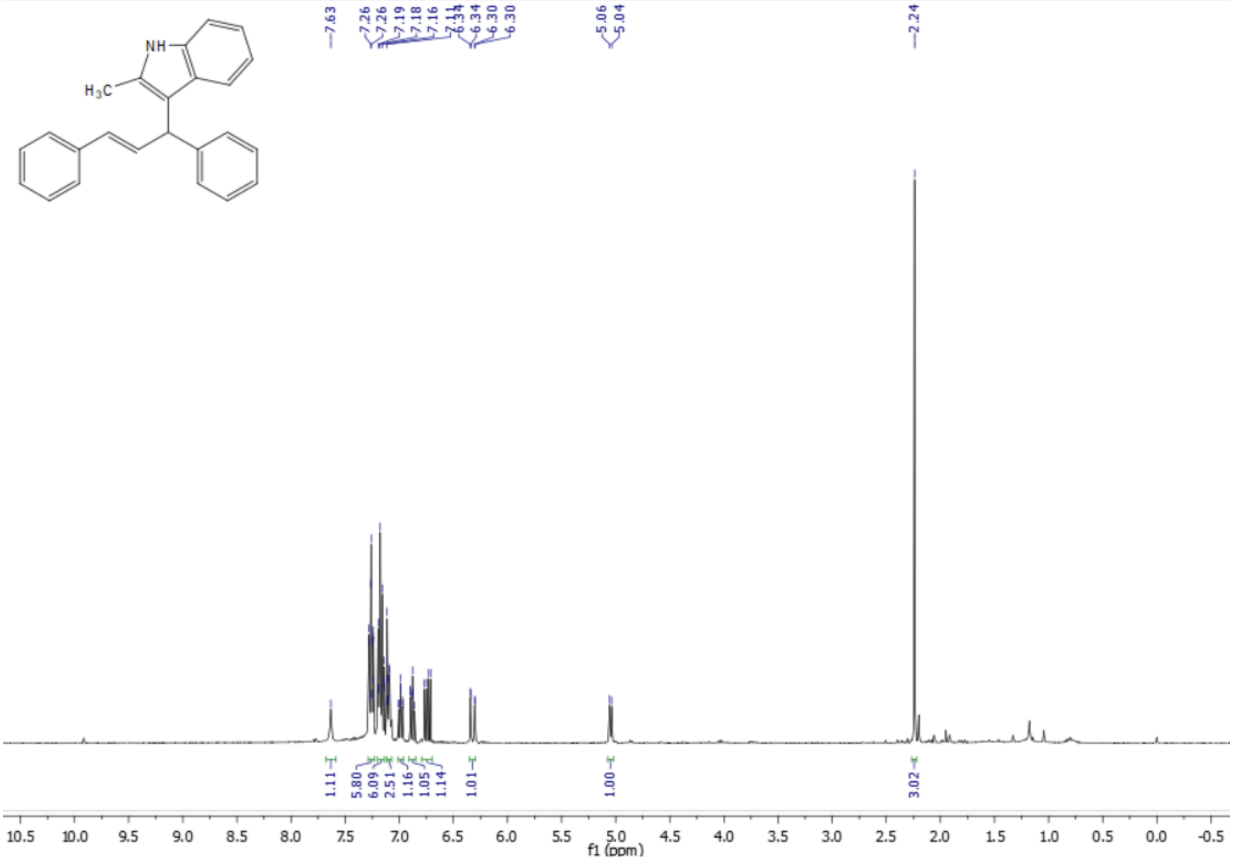

Figure S26. ^{1}H NMR spectrum of (*E*)-3-(1,3-diphenylallyl)-2-methyl-1*H*-indole (3)

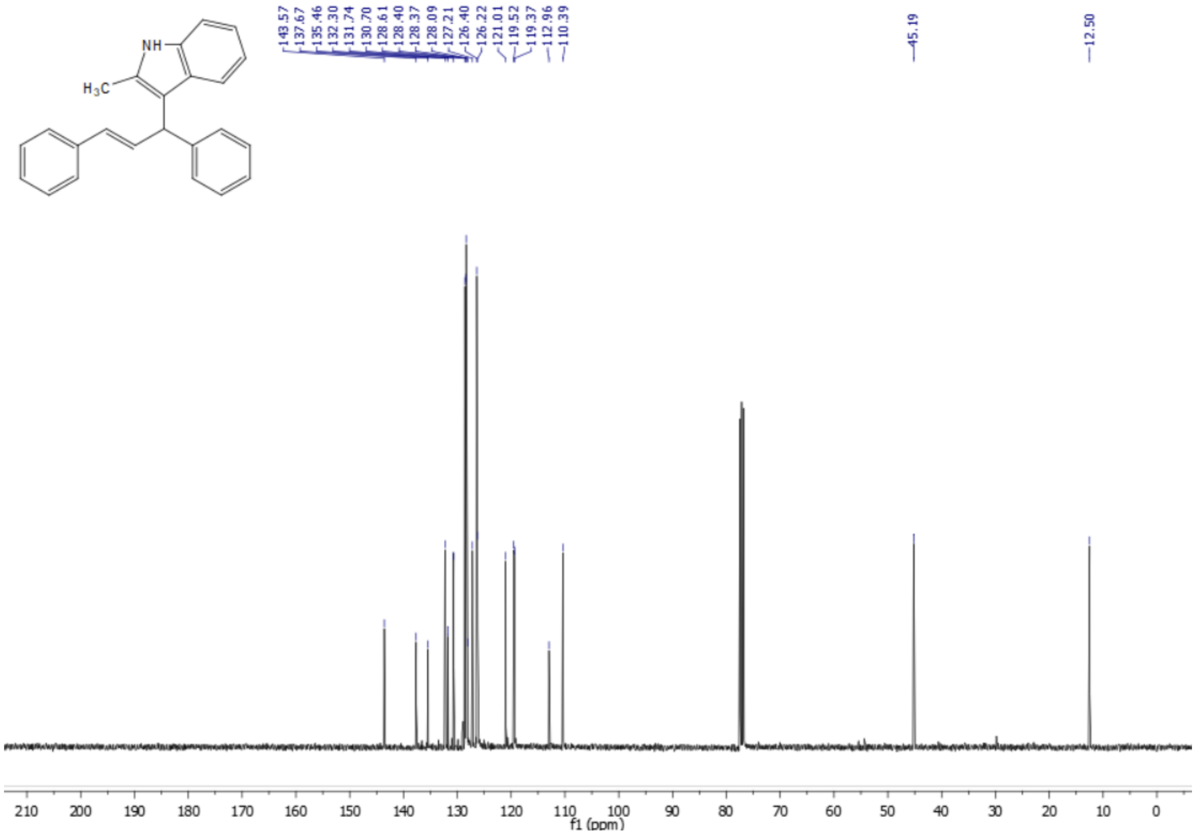

Figure S27. ^{13}C NMR spectrum of (*E*)-3-(1,3-diphenylallyl)-2-methyl-1*H*-indole (3)

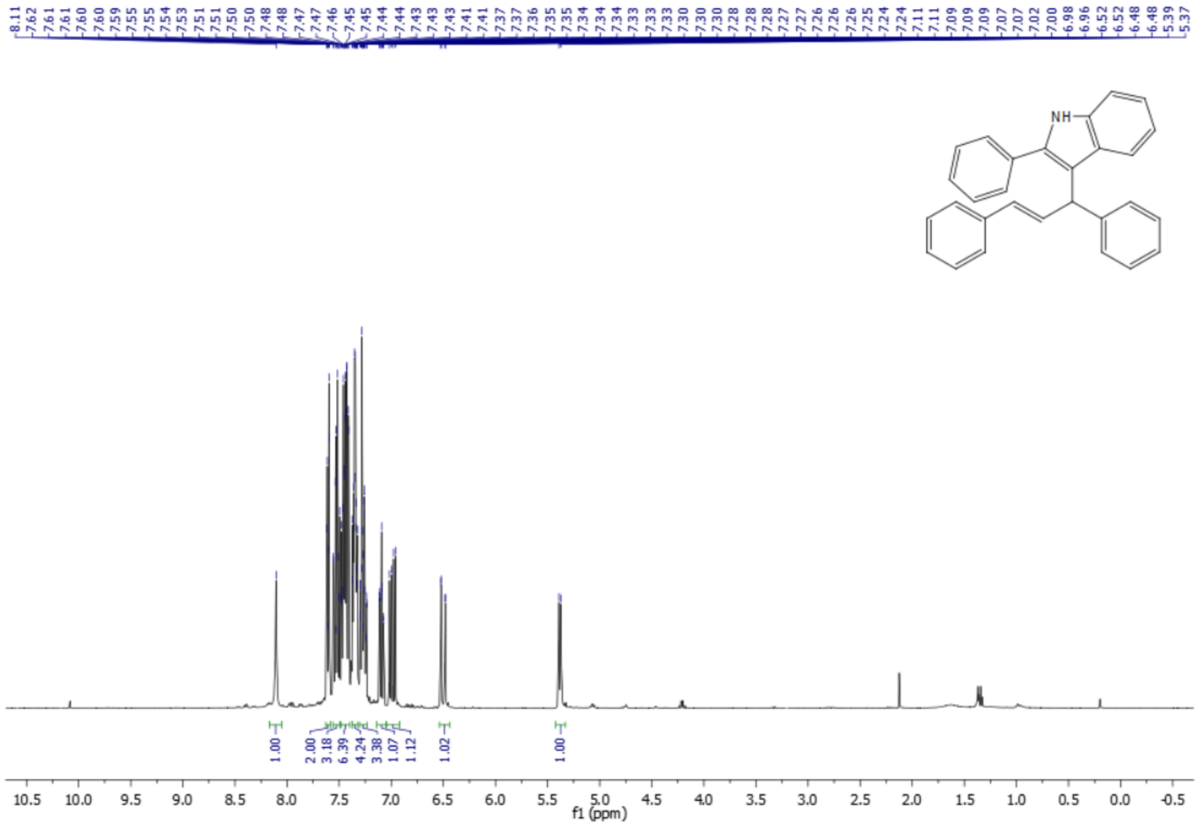

Figure S28. ^{1}H NMR spectrum of (*E*)-3-(1,3-diphenylallyl)-2-phenyl-1*H*-indole (4)

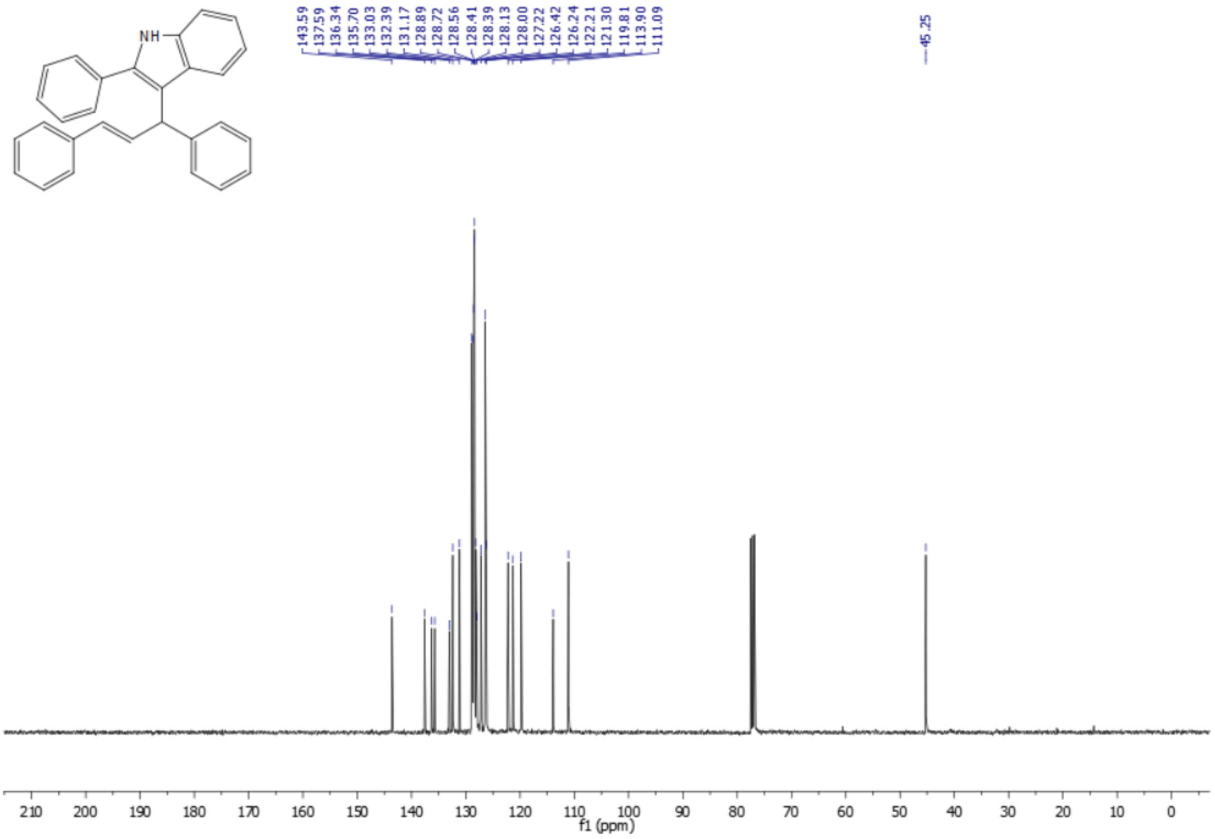

Figure S29. ^{13}C NMR spectrum of (*E*)-3-(1,3-diphenylallyl)-2-phenyl-1*H*-indole (4)

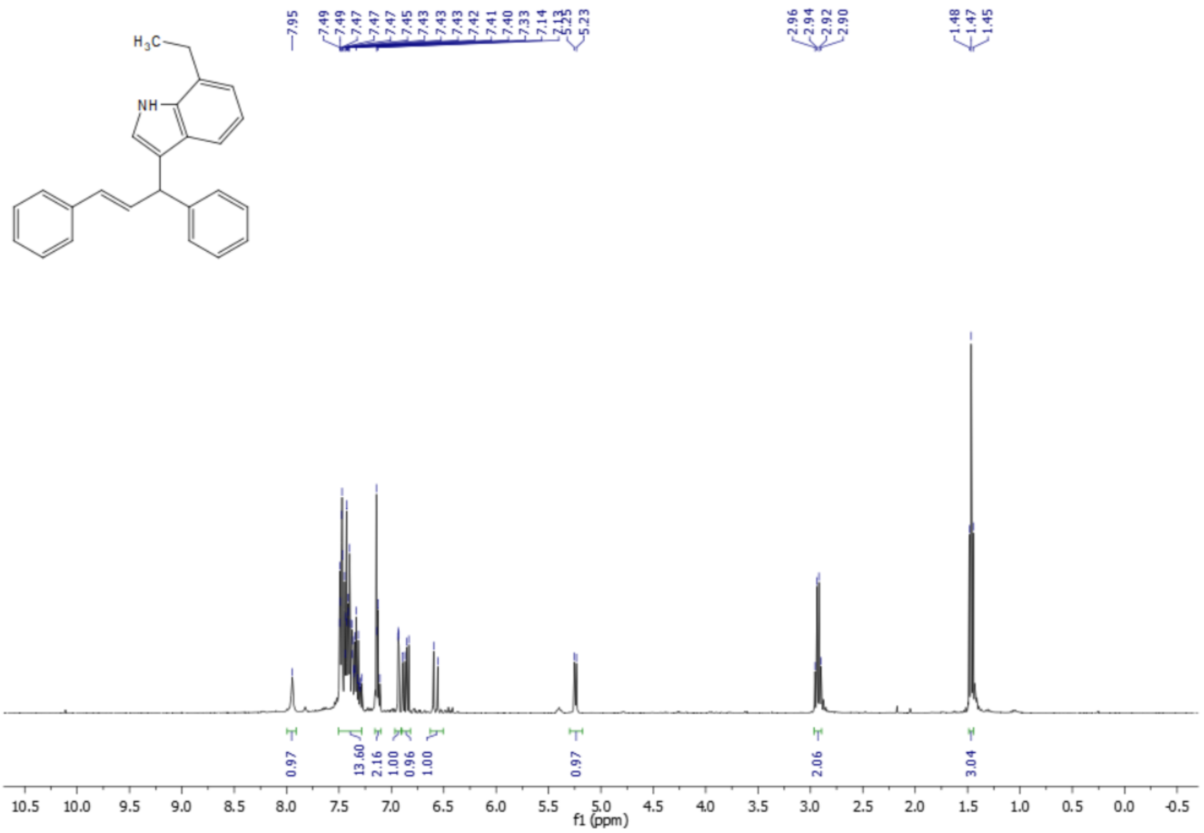

Figure S30. ^{1}H NMR spectrum of (*E*)-3-(1,3-diphenylallyl)-7-ethyl-1*H*-indole (5)

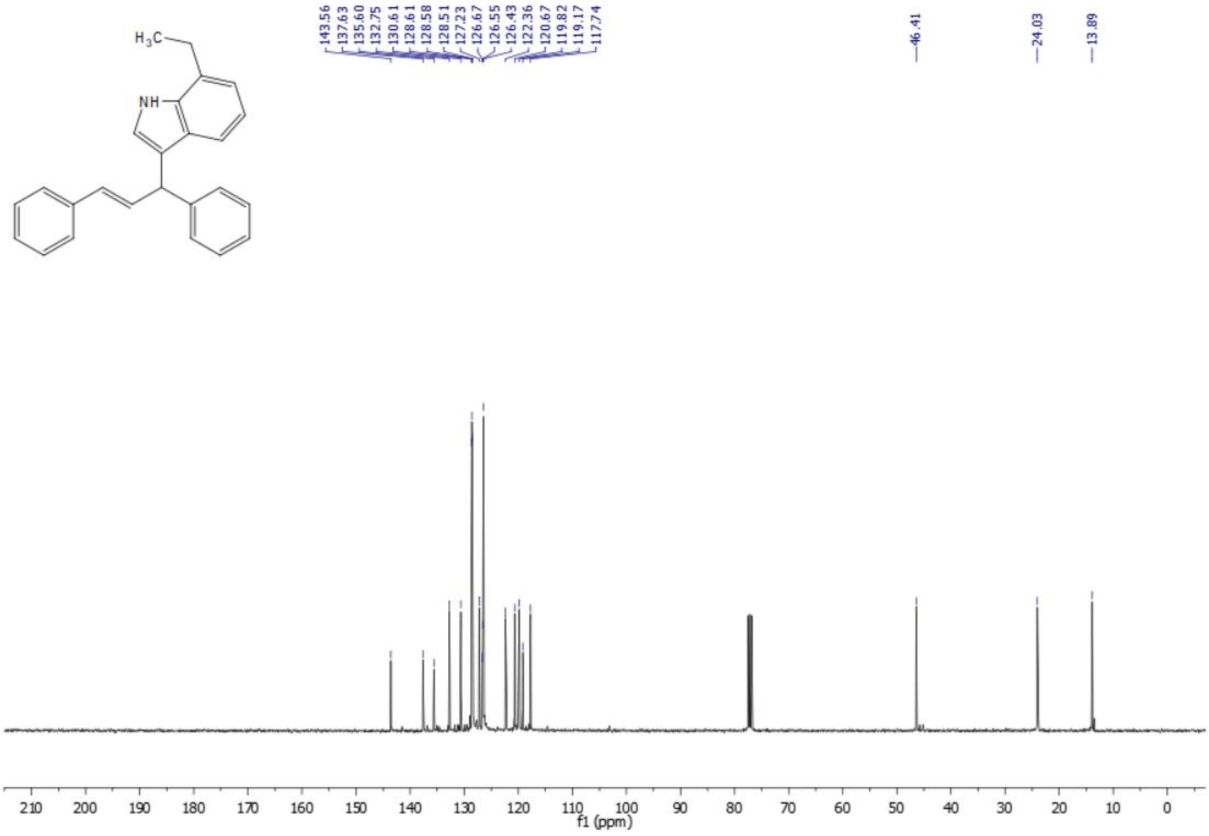

Figure S31. ^{13}C NMR spectrum of (*E*)-3-(1,3-diphenylallyl)-7-ethyl-1*H*-indole (5)

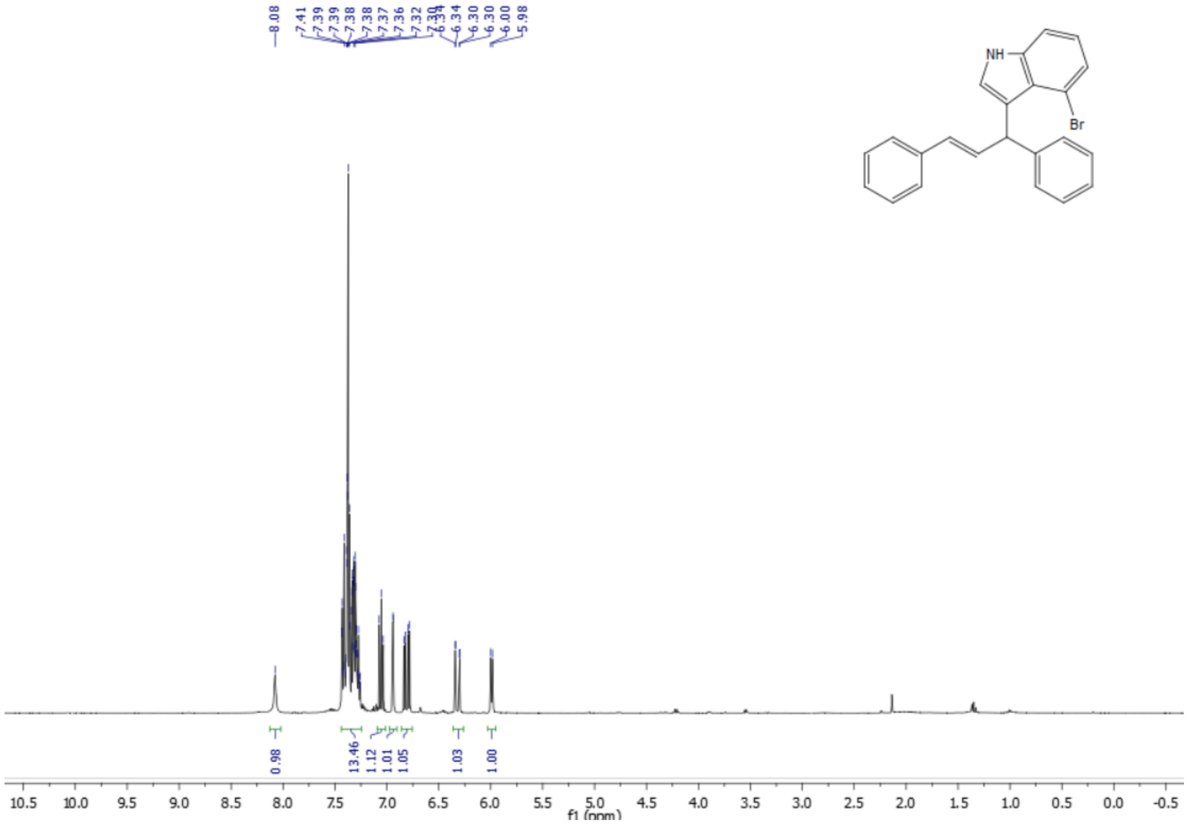

Figure S32. ^{1}H NMR spectrum of (*E*)-4-bromo-3-(1,3-diphenylallyl)-1*H*-indole (6)

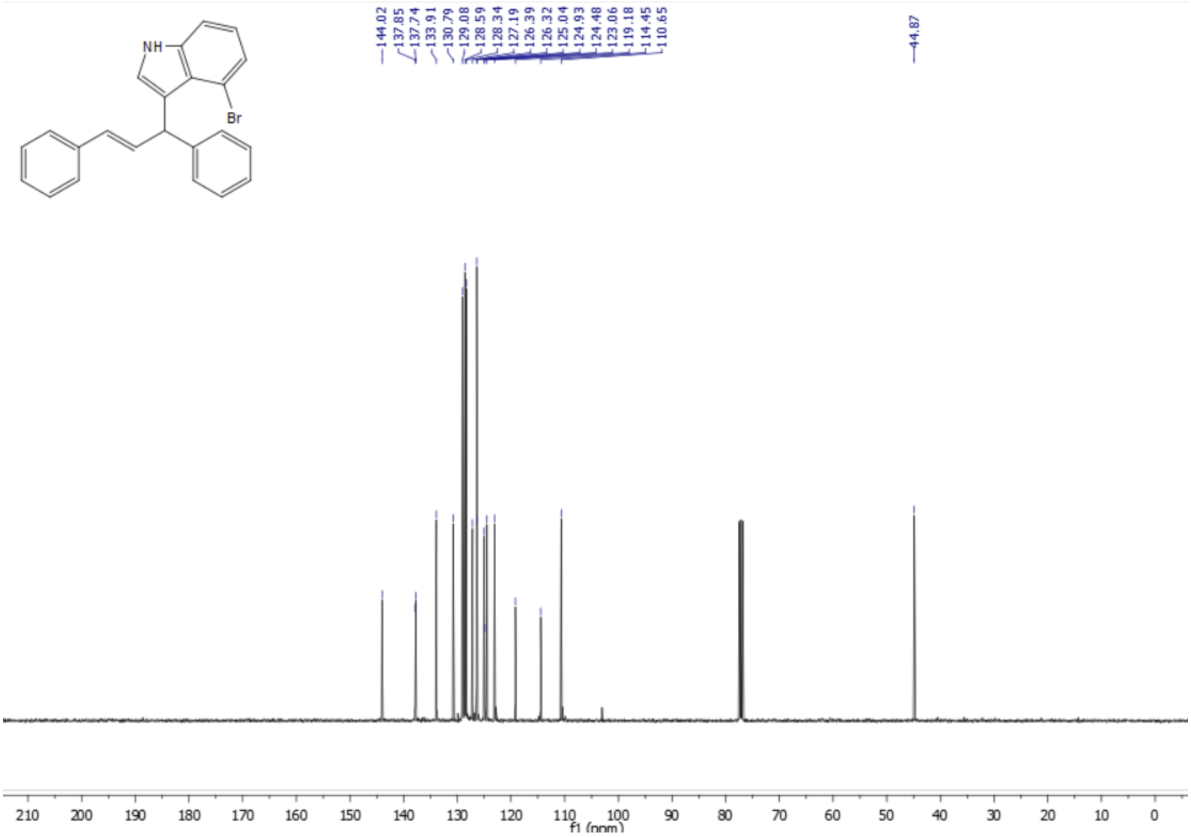

Figure S33. ^{13}C NMR spectrum of (*E*)-4-bromo-3-(1,3-diphenylallyl)-1*H*-indole (6)

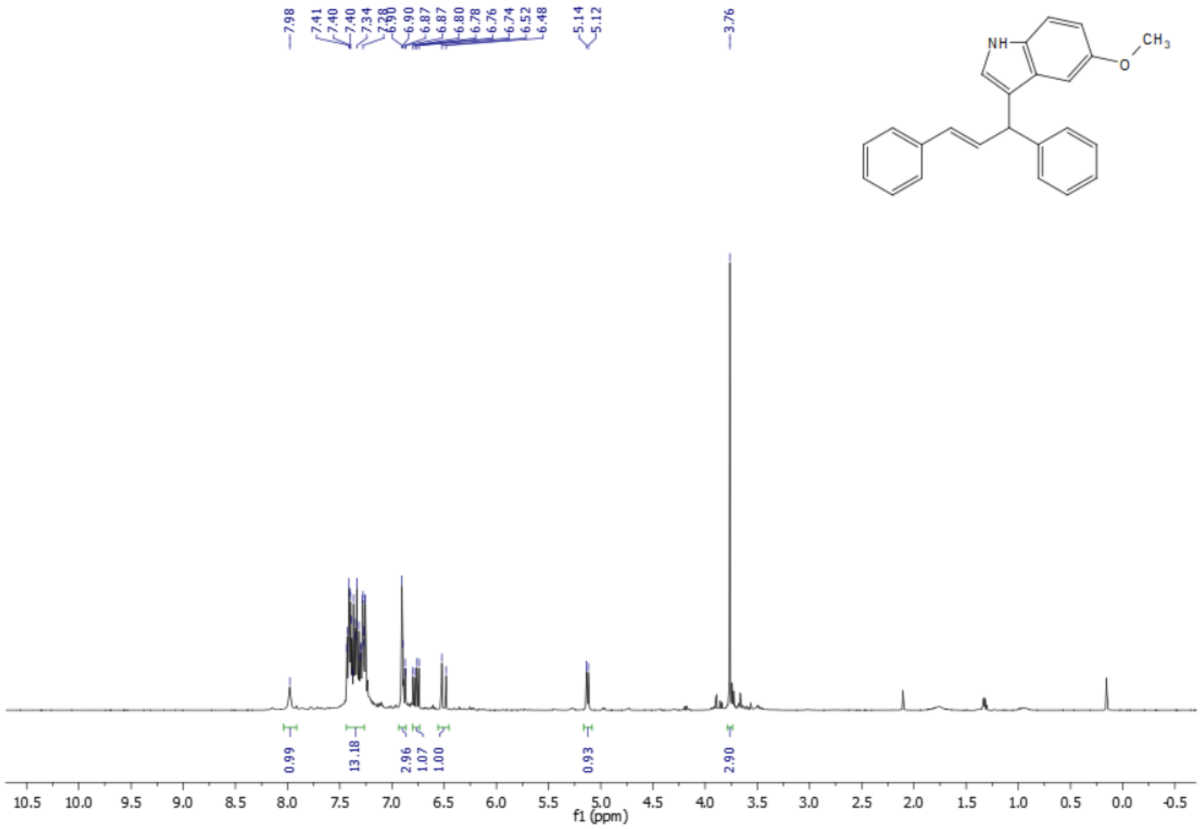

Figure S34. ^{1}H NMR spectrum of (*E*)-3-(1,3-diphenylallyl)-5-methoxy-1*H*-indole (7)

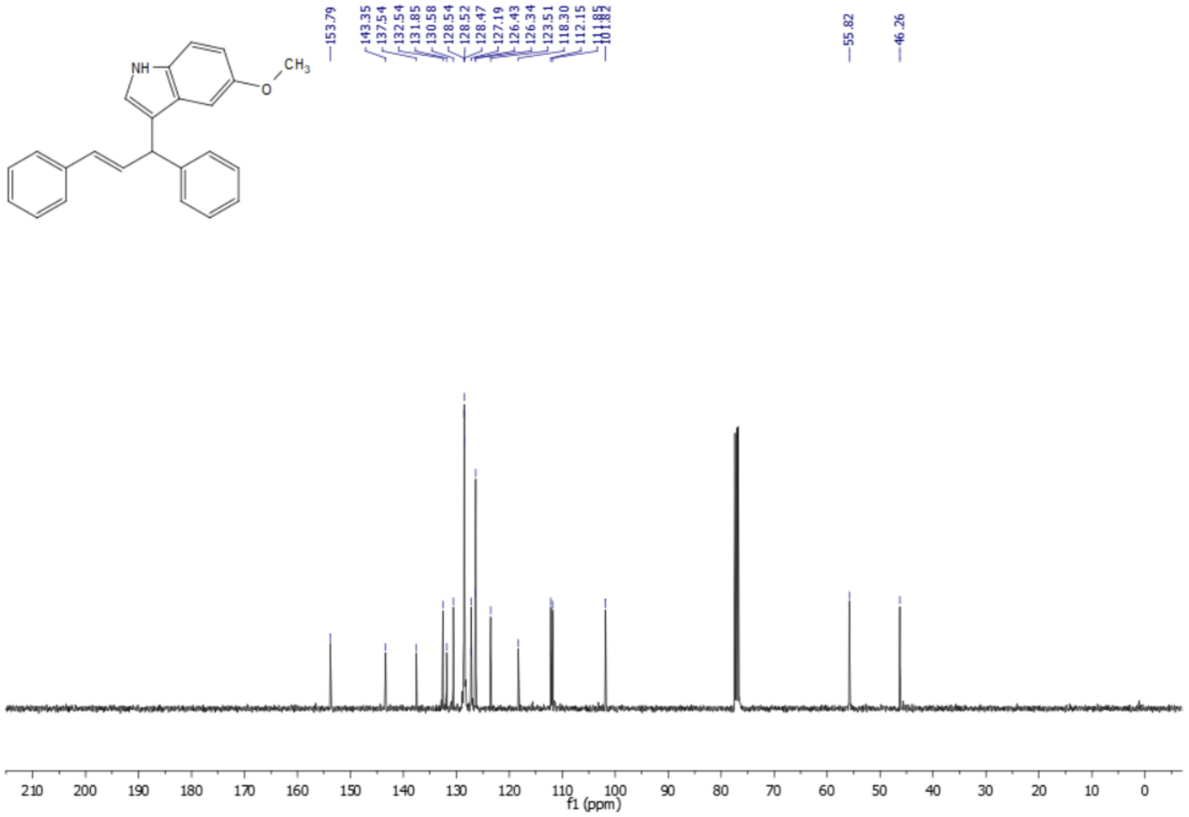

Figure S35. ^{13}C NMR spectrum of (*E*)-3-(1,3-diphenylallyl)-5-methoxy-1*H*-indole (7)

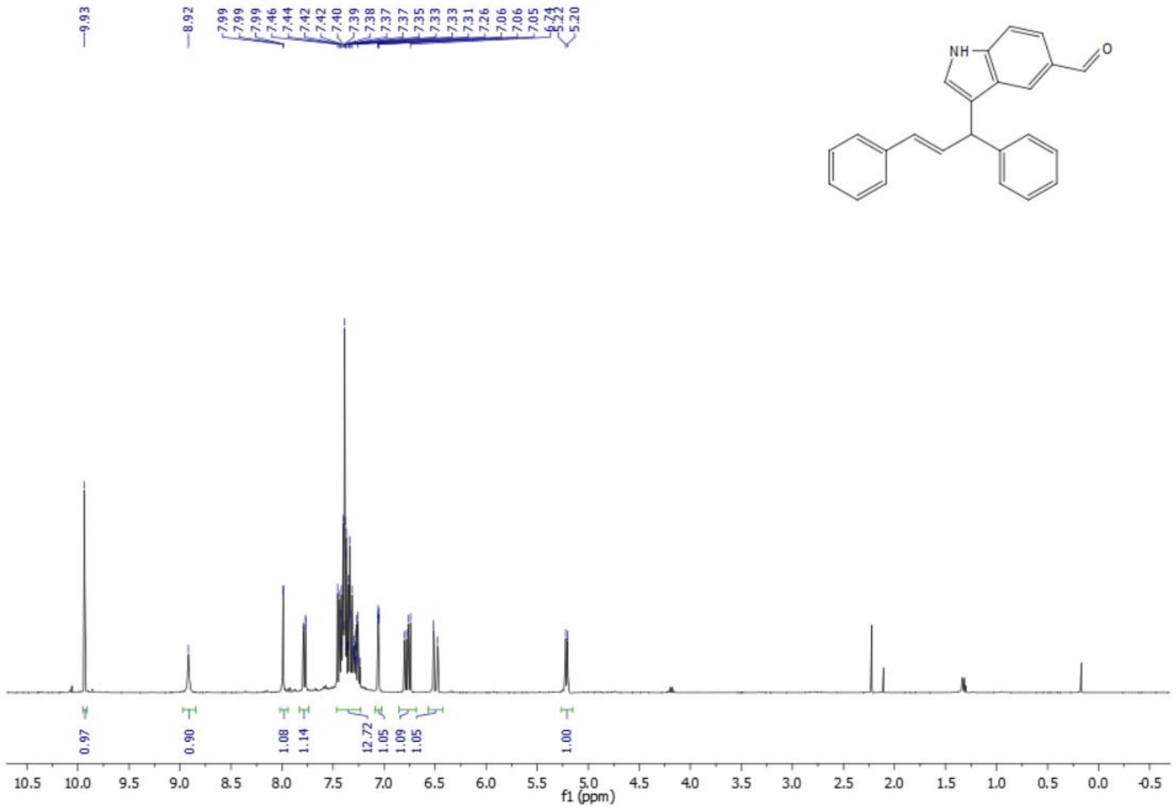

Figure S36. ^{1}H NMR spectrum of (*E*)-3-(1,3-diphenylallyl)-5-formyl-1*H*-indole (8)

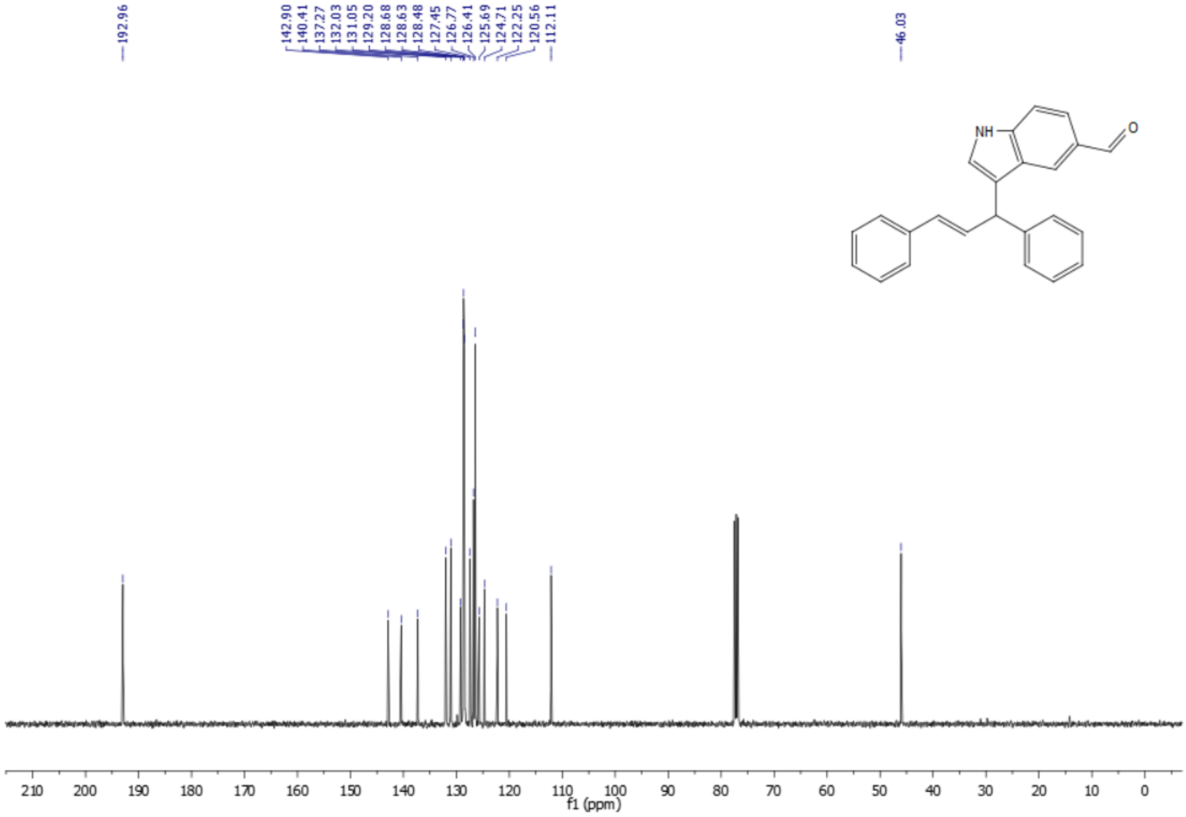

Figure S37. ^{13}C NMR spectrum of (*E*)-3-(1,3-diphenylallyl)-5-formyl-1*H*-indole (8)

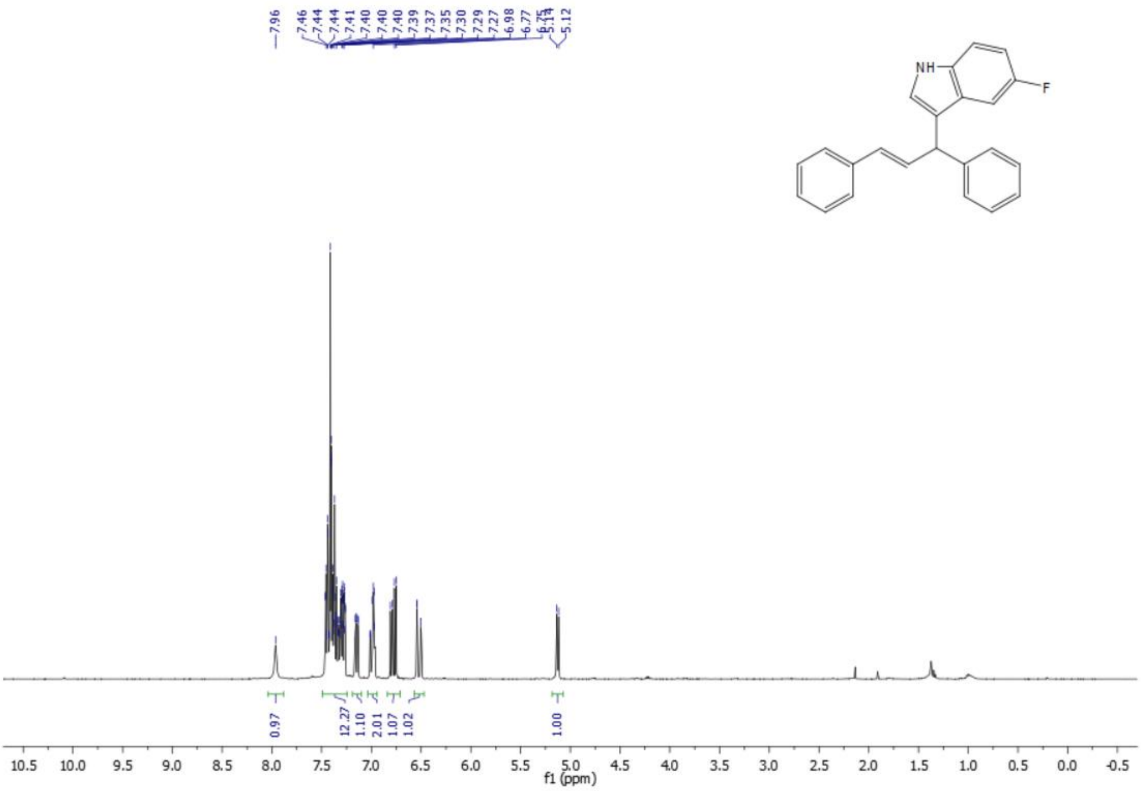

Figure S38. ¹H NMR spectrum of (*E*)-3-(1,3-diphenylallyl)-5-fluoro-1*H*-indole (9)

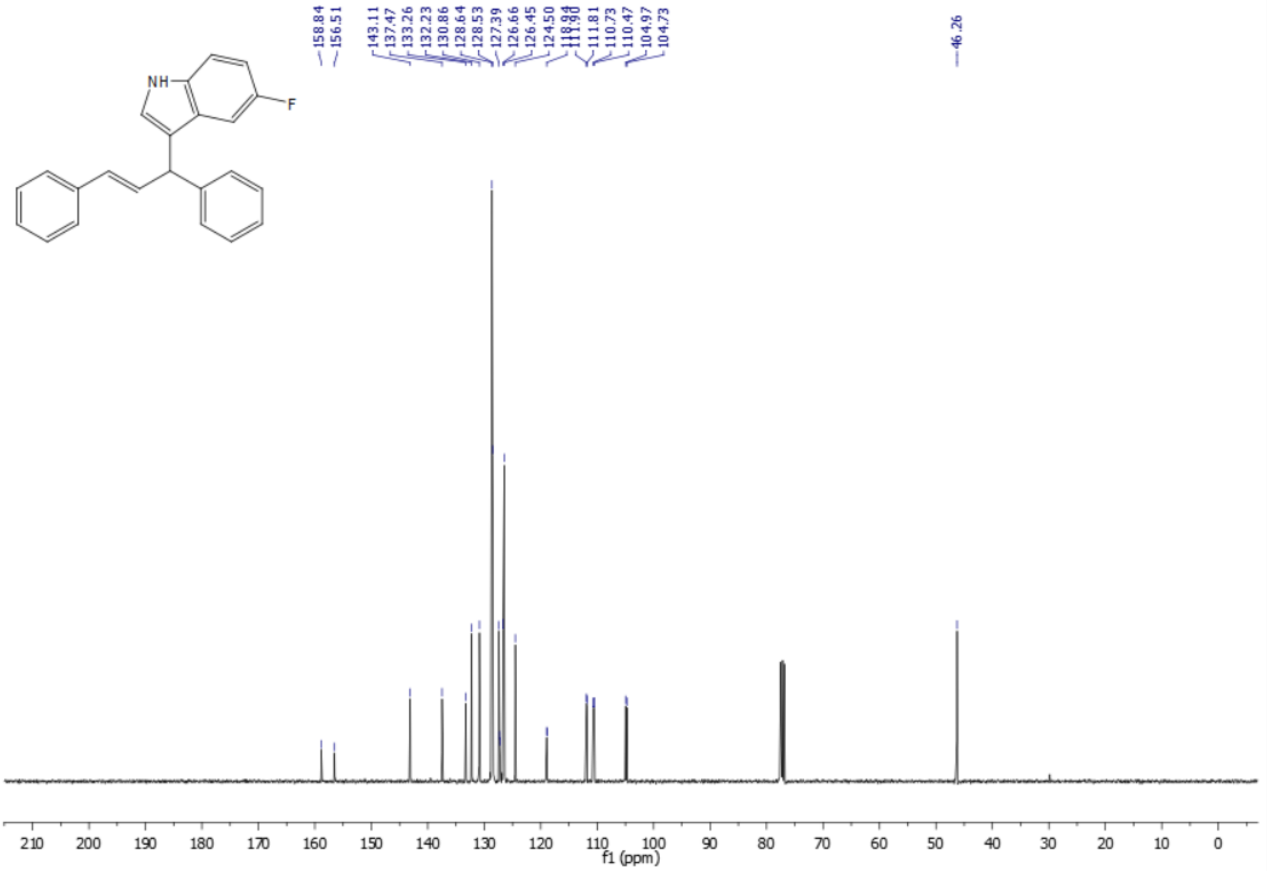

Figure S39. ¹³C NMR spectrum of (*E*)-3-(1,3-diphenylallyl)-5-fluoro-1*H*-indole (9)

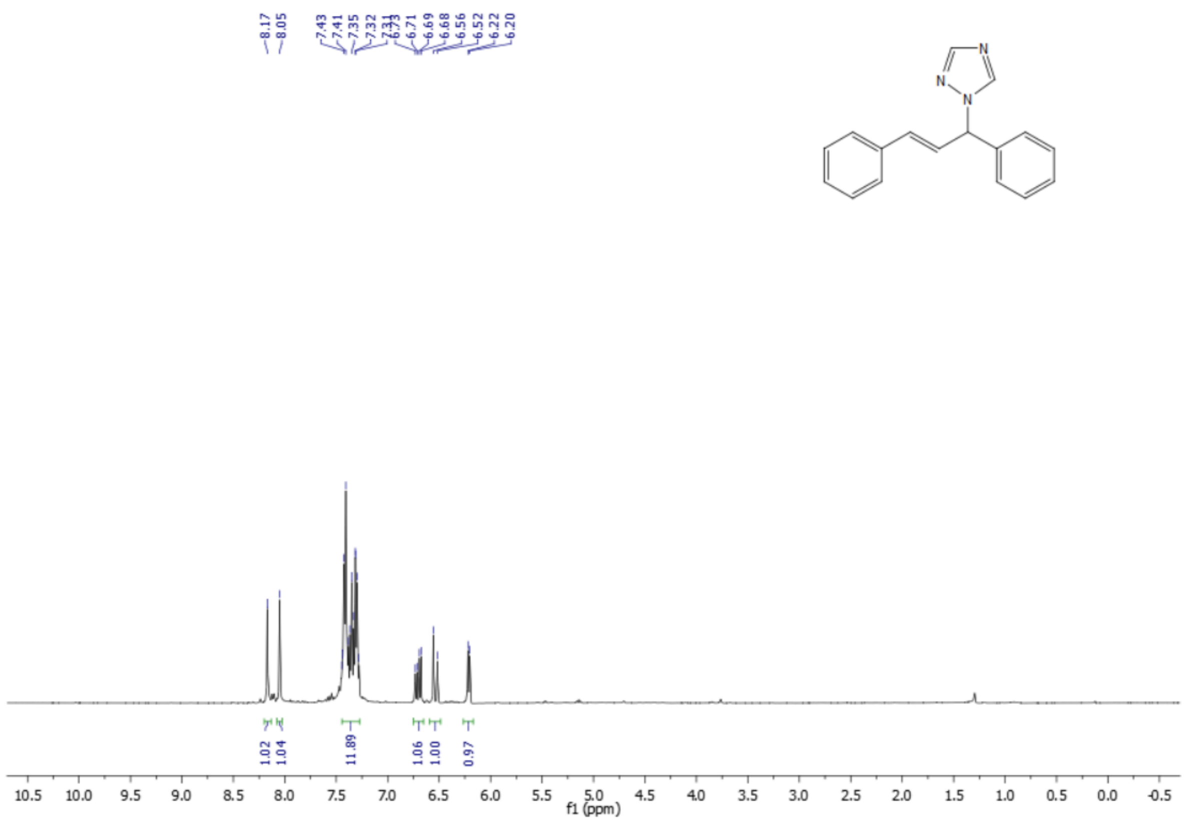

Figure S40. ^{1}H NMR spectrum of (*E*)-1-(1,3-diphenylallyl)-1,2,4-triazole (10)

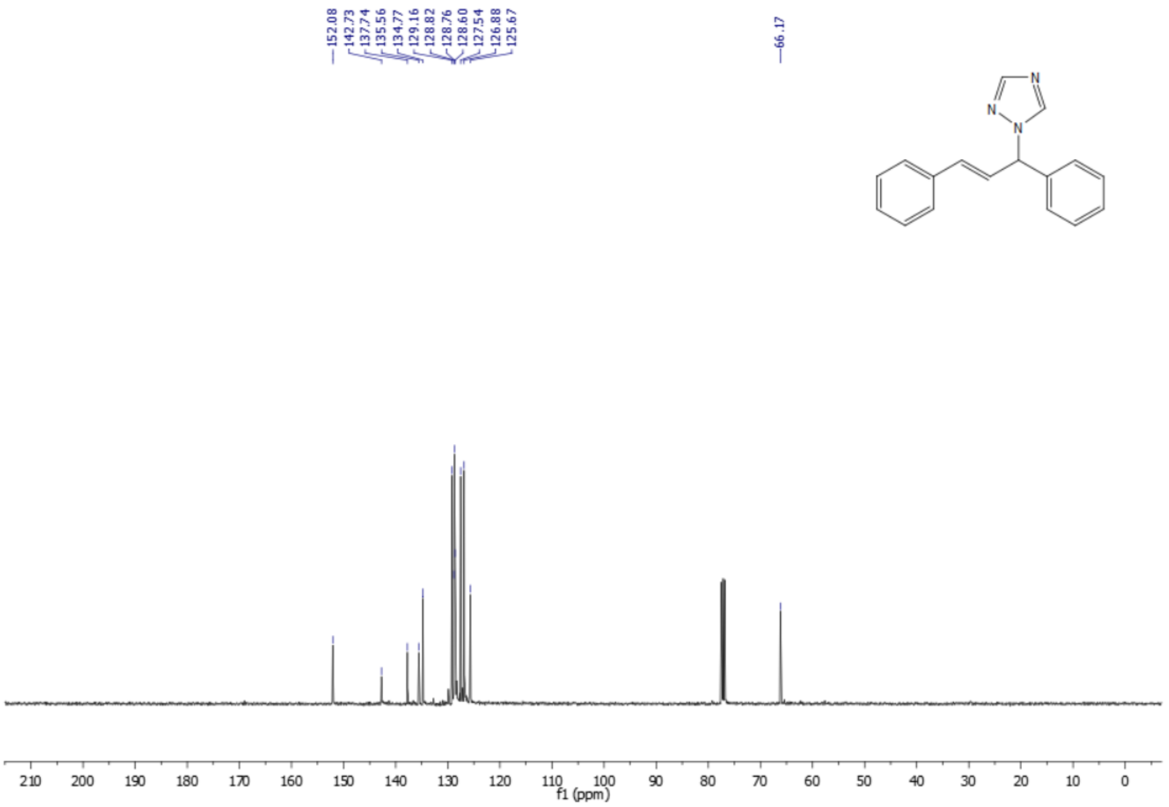

Figure S41. ^{13}C NMR spectrum of (*E*)-1-(1,3-diphenylallyl)-1,2,4-triazole (10)

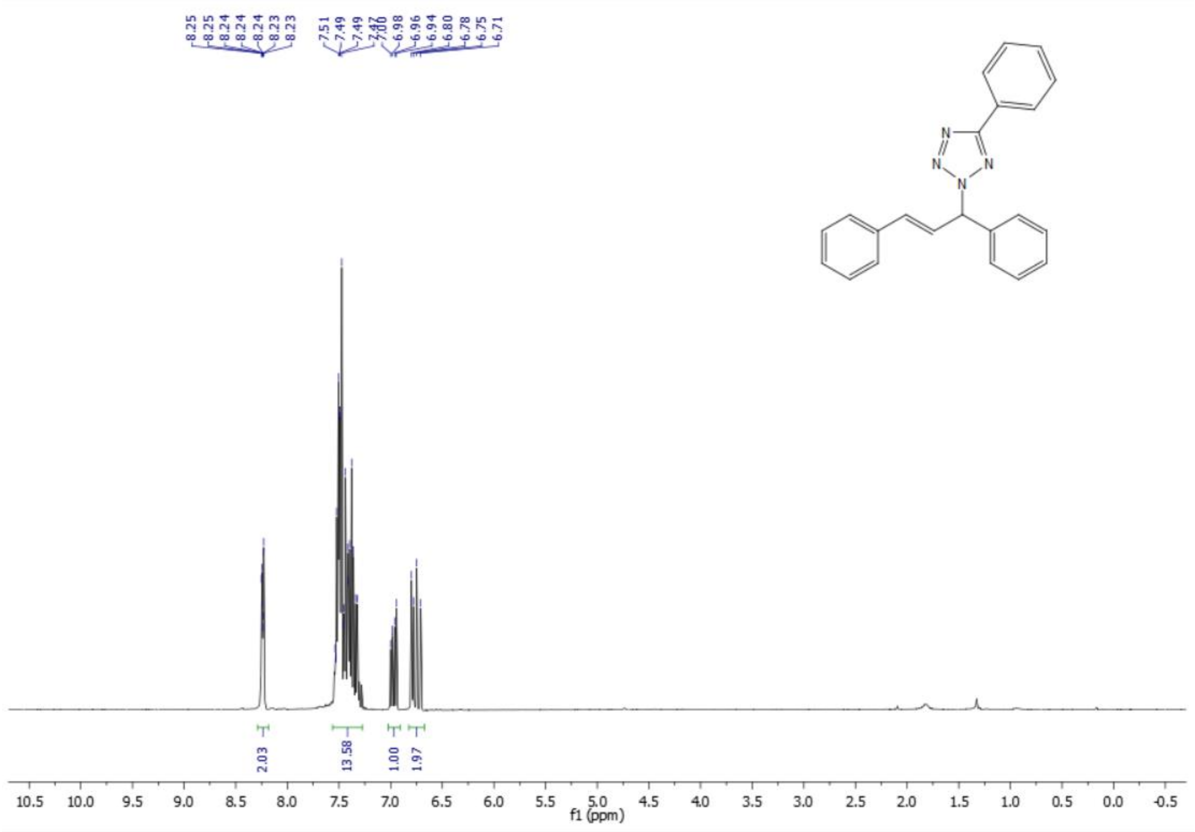

Figure S42. ^{1}H NMR spectrum of (*E*)-2-(1,3-diphenylallyl)-5-phenyl-2*H*-tetrazole (11)

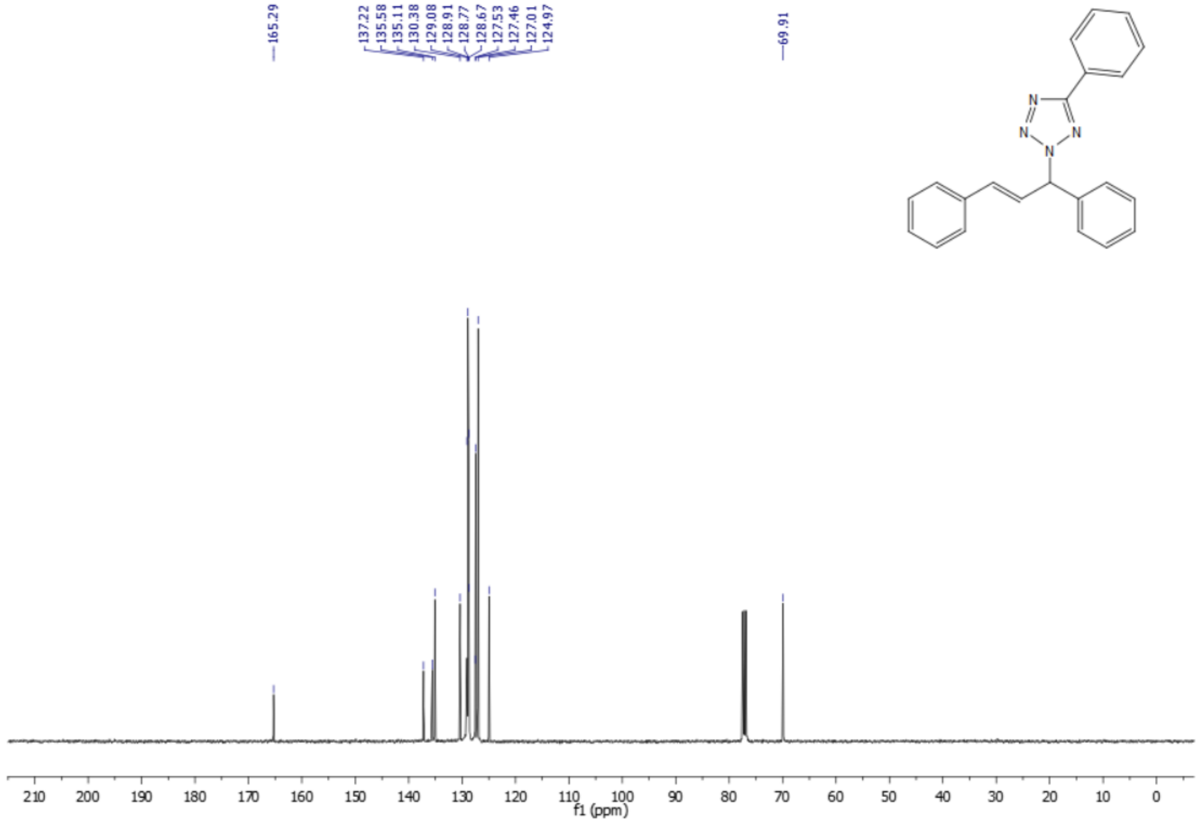

Figure S43. ^{13}C NMR spectrum of (*E*)-2-(1,3-diphenylallyl)-5-phenyl-2*H*-tetrazole (11)

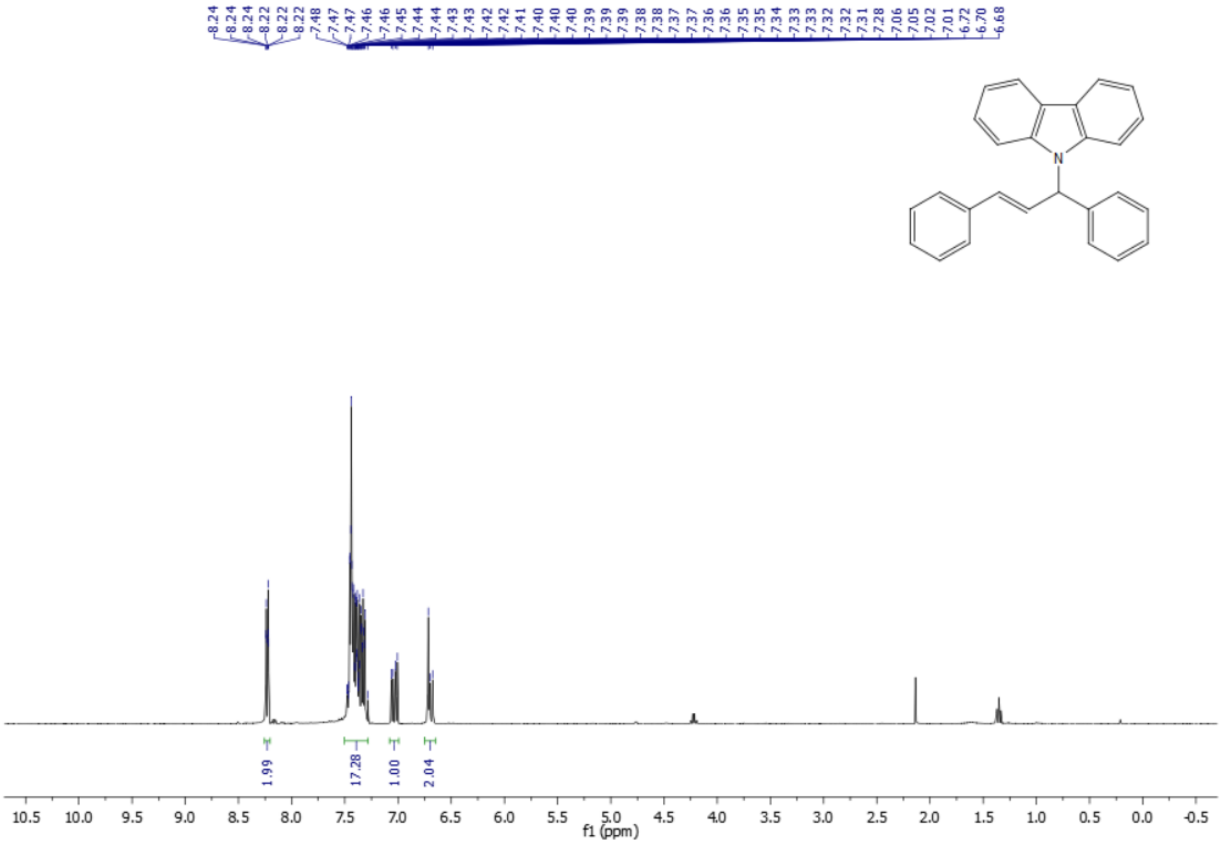

Figure S44. ¹H NMR spectrum of (*E*)-9-(1,3-diphenylallyl)-9*H*-carbazole (12)

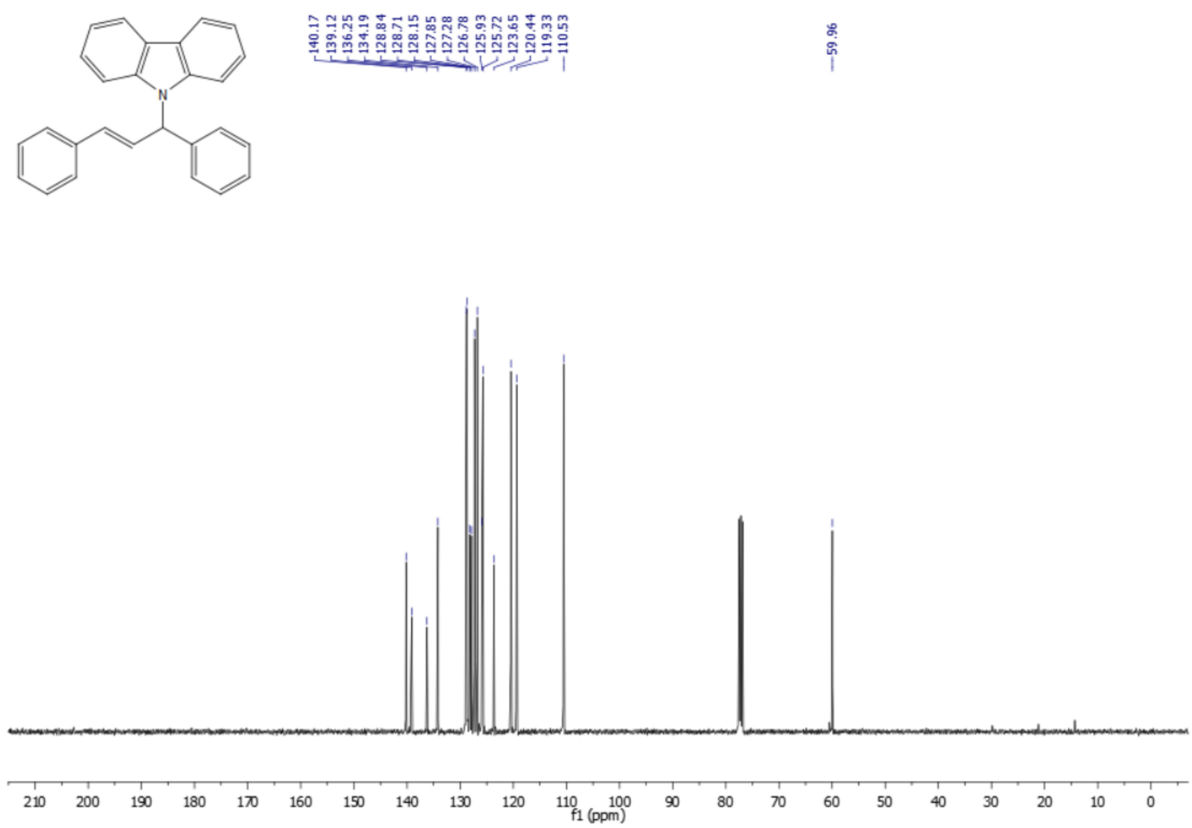

Figure S45. ¹³C NMR spectrum of (*E*)-9-(1,3-diphenylallyl)-9*H*-carbazole (12)

Miscellaneous images

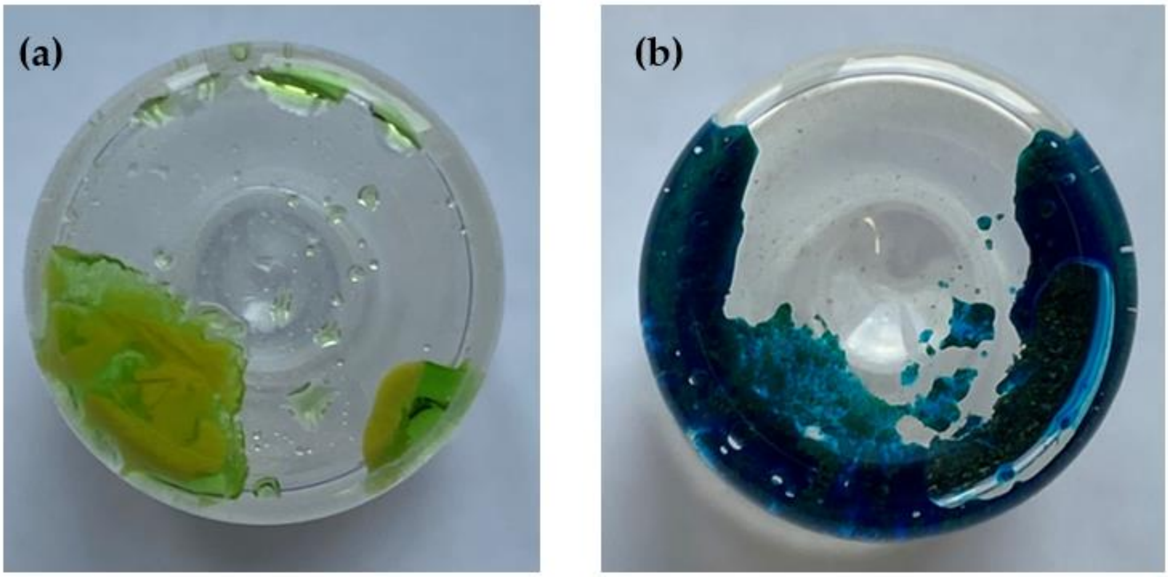

Figure S46. 1:1 mcmimCl:$NiCl_2 \cdot 6H_2O$ mixture (a) cooled down for 5 minutes after preparation (b) heated up again until development of condensation
